# Cryo-EM structure of soluble VPS13C suggests its regulation by a conformational switch and by calmodulin

**DOI:** 10.1016/j.molcel.2026.06.028

**Published:** 2026-07-07

**Authors:** Dazhi Li, Xinbo Wang, Hongyan Hao, Jessica Eden, Bodan Hu, Emma E. Walsh, Matthew A. H. Parson, Stephanie Hamill, Yuting Li, Guochao Chen, John E. Burke, Pietro De Camilli, Karin M. Reinisch

**Affiliations:** 1 Department of Cell Biology, Yale University School of Medicine, New Haven, CT, USA.; 2 Aligning Science Across Parkinson’s (ASAP) Collaborative Research Network, Chevy Chase, MD, USA.; 3 Department of Neuroscience, Yale University School of Medicine, New Haven, CT, USA.; 4 Program in Cellular Neuroscience, Neurodegeneration, and Repair, Yale University School of Medicine, New Haven, CT, USA.; 5 Howard Hughes Medical Institute, Yale University School of Medicine, New Haven, CT, USA.; 6 Department of Biochemistry and Microbiology, University of Victoria, Victoria, British Columbia, Canada; 7 University of Victoria Genome BC Proteomic Centre, Victoria, British Columbia, Canada; 8 Department of Biochemistry and Molecular Biology, The University of British Columbia, Vancouver, British Columbia, Canada

## Abstract

Bridge-like lipid transfer proteins (BLTPs) play fundamental roles in cellular lipid redistribution between organellar membranes. They comprise bridge-domains spanning between organelles at contact sites that allow lipids to transit the cytosol between adjacent membranes. The assembly of BLTPs into complexes with adaptor proteins enables lipid transfer. To address the mechanisms underlying assembly and regulation of BLTP complexes, we used cryo-EM to resolve the structure of one such BLTP, the Parkinson’s protein VPS13C, at near-atomic resolution. The structure identifies a lipid-transfer-nonpermissive conformation, where the built-in C-terminal VAB adaptor module blocks the end of the lipid transfer bridge, interfering with lipid delivery. We also identify calmodulin, central to calcium signaling, as a constitutive VPS13C interactor. Calcium induces conformational changes in VPS13C-CaM, suggesting calcium regulation of VPS13 function. Altogether, this structure of intact VPS13C serves as starting point to understand its regulation and that of other VPS13 proteins.

## eToc

VPS13C, whose dysfunction causes Parkinson’s disease, adopts a lipid-transfer inactive conformation in the cytoplasm, and relies on calmodulin for proper folding and localization to membrane surfaces.

## Graphical Abstract

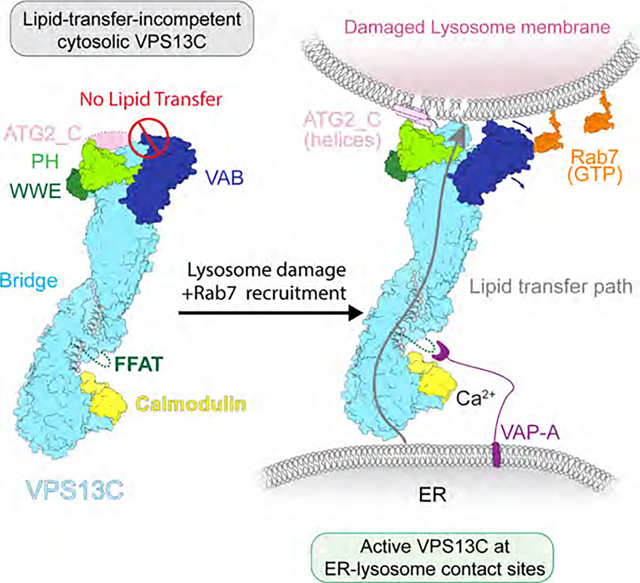

## Introduction

In eukaryotic cells, the lipids comprising organellar membranes are synthesized mainly in the endoplasmic reticulum (ER) and then redistributed from there to other organelles. While vesicle trafficking is long known as a mechanism for bulk lipid transfer between organelles, the fundamental role of very large (~200–575 kDa) rod-like proteins, so-called bridge-like lipid transport proteins (BLTPs), is only recently recognized.^1–3^ These proteins can span between the ER and other organelles at contact sites, where the organelles are closely apposed, via “bridge” domains. Such domains feature hydrophobic grooves that allow lipids to travel through the cytosol between organellar membranes. Lipid transfer via BLTP underlies multiple functions, including membrane expansion, as required for the biogenesis of new organelles such as the autophagosome, or the maintenance of other organelles, such as mitochondria and peroxisomes which are disconnected from vesicle trafficking pathways and so rely exclusively on protein-mediated transport for their membrane lipid supply.

Several recent studies have shown that BLTPs do not work alone but rather in complexes with partner proteins^1,2,4^ that can include adaptors to anchor the tips of the bridge domain to donor and acceptor organelles, as well as integral membrane proteins that facilitate the transfer or redistribution of lipids at the BLTP-membrane junction. Identification of partner proteins and mechanisms that control their assembly with VPS13, including potential conformational changes of VPS13, are subject of ongoing investigations and prerequisite for understanding the molecular mechanisms underlying BLTP-mediated lipid transfer and its regulation.

**Figure 1.**
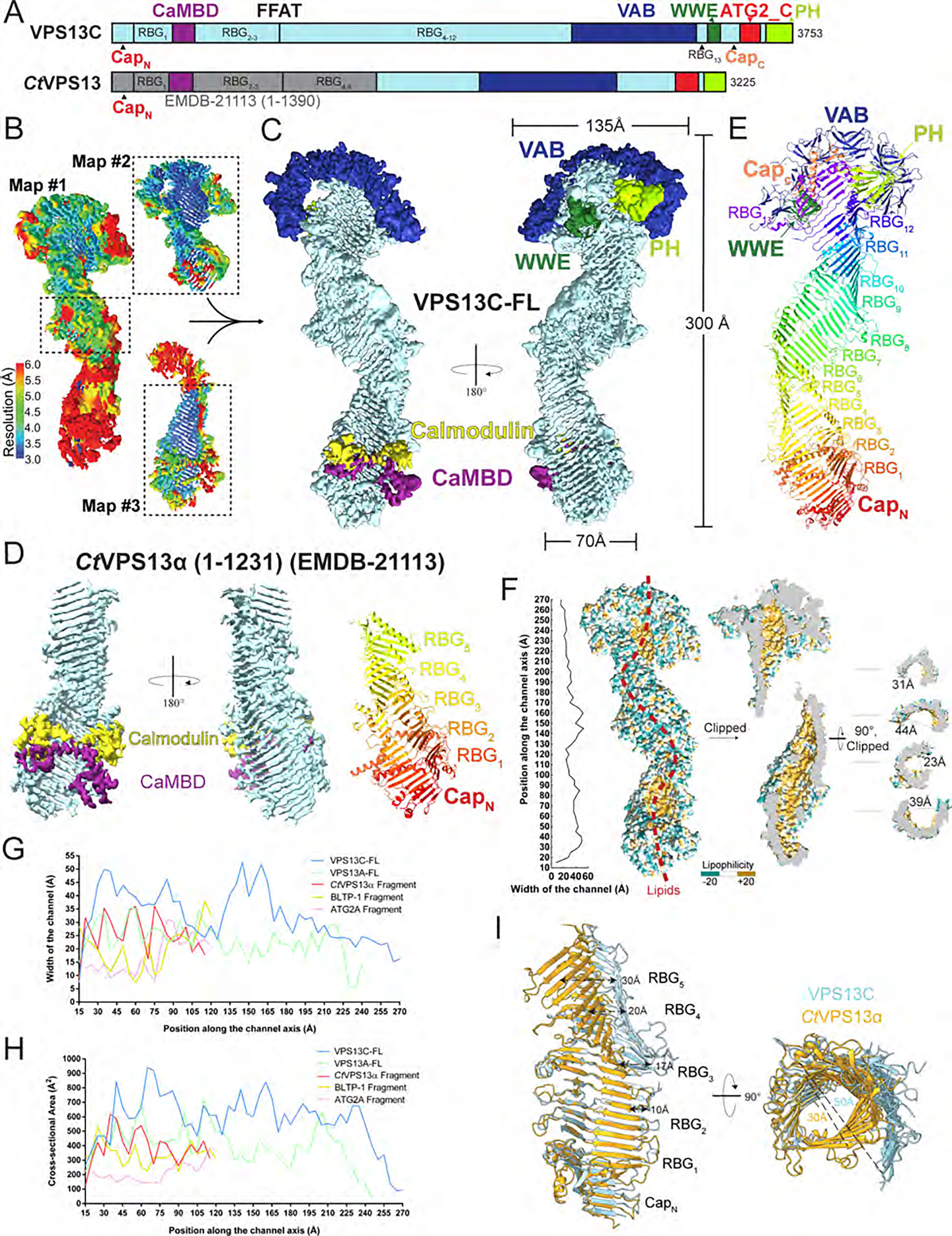
Structural analysis of VPS13C and *Ct*VPS13α in complex with CaM. (**A**) Domain architecture of human VPS13C and *Ct*VPS13α. (**B**) For VPS13C, locally-refined maps #2 and #3 are combined with the central portion of the full-length map (#1) to generate a composite map. Maps are colored according to local resolution (see also Fig. S2, S3, Table 1). (**C**) Composite map of VPS13C-CaM showing the bridge domain (light blue), C-terminal VAB- (dark blue), PH- (light green), and WWE- (dark green) domains, and N-terminal CaM-binding domain (CaMBD, purple), which binds CaM (yellow). (**D**) Cryo-EM map (EMDB-21113) and model (residues 1–1231) of the N-terminal fragment of *Ct*VPS13α indicating CaMBD and co-purified CaM. The N-terminal “cap” and five individual RBG domains are in different colors. € Model of VPS13C alone, with different colors for the terminal “caps”, thirteen RBG domains, and adaptor domains. Dashed lines indicate flexible loops unresolved in maps. (**F**) VPS13C harbors a hydrophobic channel with varying width. Representative cross-sections are shown at right. The red dotted line indicates the presumed lipid transfer path. (**G, H**) Comparison of channel width and the cross-sectional area along the channel axis in different experimental structures of BLTPs, calculated as for (F). (**I**) Overlay of *Ct*VPS13α with VPS13C’s N-terminal region, including the “cap” and the first five RBG motifs. For clarity, only RBG ß-strands are shown.

As founding members of the BLTP super family, VPS13 proteins are among the best studied, including by our groups^1,2^. They are conserved across eukaryotes, with a single protein in yeasts and four versions (A-D) in humans. The VPS13s feature built-in adaptor modules at the bridge domain’s C-terminal end, which mediate their association with the lipid acceptor organelle (Fig. 1A). These include amphipathic helices in the ATG2_C region (so-called because such helices are also present in ATG2 proteins, another BLTP family) the PH- and VAB domains, as well as a WWE-domain in VPS13A and -C (Fig 1A). In human VPS13s (except VPS13B), a peptide (called “FFAT”) motif near the N-terminal end of the bridge-domain mediates their association with the lipid donor organelle, the ER, via an interaction with the ER-resident VAP.^5^ The identity of other proteins that might couple the N-terminal end of VPS13 to the donor organelle had heretofore been elusive.

In humans, VPS13 dysfunction is associated with severe neurological diseases^1^, including chorea acanthocytosis for VPS13A^6,7^ and an early onset version of Parkinson’s for VPS13C^8^, making them of significant biomedical interest. Whereas the single yeast VPS13 multi-tasks, each of the human VPS13s has distinct localizations and functions in the cell^1^, explaining their different disease manifestations. VPS13s play roles in mitochondrial maintenance; in the formation of autophagosome-, prospore (in yeast)-, and acrosomal membranes; in peroxisome biogenesis; and in Golgi homeostasis.^2^ VPS13C, in particular, plays a key role in lysosome homeostasis as it is localized at contacts between the ER and late endosomes and lysosomes (hence referred to as lysosomes). This localization is highly regulated as VPS13C is massively recruited to these contacts in response to lysosomal damage^9^, presumably to deliver lipids for repair of the lysosome membrane.

Although first insights into the structure of VPS13 proteins were obtained from x-ray and cryo-electron microscopy (cryo-EM) studies of VPS13 fragments^5,10^ and complemented by AlphaFold predictions^3^, our understanding of their mechanism of action and regulation has been hindered by the lack of experimental structural information for any intact protein, especially pertaining to the disposition of the adaptor domains with respect to the bridge domain. Here, to obtain such insights, we used cryo-EM to visualize an intact VPS13, the Parkinson’s protein VPS13C (425 kDa), at near atomic resolution (in 3 maps, 3.8–4.2 Å). The maps featured density close to the N-terminal tip of the bridge domain that could not be accounted for by VPS13C alone, and which we subsequently identified as belonging to calmodulin (CaM), which co-purifies with VPS13s in a high affinity complex. This finding raises the possibility that CaM may participate in controlling VPS13 recruitment and/or attachment to the lipid donor membrane and, moreover, highlights growing evidence for calcium signaling in regulating VPS13 activity. At VPS13C’s C-terminal end, the reconstruction shows the VAB domain arching over the bridge-domain to interfere with its binding to the acceptor membrane and hence with lipid transfer. Thus, purified VPS13C, likely representing the cytosolic form of VPS13C not engaged at membrane contact sites, is in a lipid-transfer incompetent state. The protein must undergo conformational changes as it engages with the acceptor membrane to allow the bridge domain to access this membrane. The occurrence of such conformational change is supported by a parallel study of another VPS13 family member, VPS13A, in a complex with the scramblase XK.^11^ As the VAB domain is a common feature of all VPS13s, they likely all feature lipid-transfer inactive and active conformations, and the transition between conformations offers means for regulating their activity.

## Results

### Cryo-EM Reconstruction.

We undertook biochemical and single particle cryo-EM studies to better understand how the function of VPS13, and particularly VPS13C, may be regulated. To this aim, we overexpressed a FLAG-tagged version of VPS13C (VPS13C-3XFLAG) in Expi293 cells and isolated it using anti-FLAG resin affinity purification, followed by size exclusion chromatography (Table S1, Fig. S1).

Electron micrographs were processed in CryoSPARC^12^ to generate 3 maps (Fig. 1B–C, Sup. Fig. S2). An initial map (#1) shows intact VPS13C at a nominal resolution of 4.2 Å, and a locally-refined map (#2) at a nominal resolution of 3.8 Å shows the C-terminal portions of VPS13C. A separate reconstruction (map #3) allowed us to visualize N-terminal portions of VPS13C at a nominal resolution of 4.1 Å. AlphaFold^13^ models guided building of VPS13C’s bridge domain, which comprises a series of 13 repeating beta groove (RBG) motifs, each consisting of 5 antiparallel beta strands, arrayed end-to-end into a taco shell shaped structure, with “caps” at the very N- and C-terminal tips of the bridge domain. We dissected the bridge-domain into RBG and “cap” segments, and placed these into the maps, then adjusted the model manually. Similarly, we placed AlphaFold models for the VAB-, PH- and WWE domains as rigid bodies, followed by manual rebuilding when appropriate. Well resolved density in map #3 allowed modeling for the N-terminal cap and RBGs 1–5, and map #2 allowed modeling of RBGs 8–13, the C-terminal “cap” and the VAB, WWE, and PH domains. RBGs 6–7 were modeled based on Map#1. There was no density corresponding to the amphipathic helices in the ATG2_C motif in any of the maps, suggesting they are unstructured, mobile, or both, in the absence of membrane. Map #3 showed density that could not be accounted for by the AlphaFold model for VPS13C, spanning over the taco shell between RBGs 1 and 2, and this density was subsequently modeled as CaM (Fig. 1C, see below).

Guided by AlphaFold^13^, we also modeled N-terminal portions of VPS13 from the fungus *Chaetomium thermophilum* (residues 2–1230), based on a previously reported single particle reconstruction at 3.8 Å nominal resolution (EMD #21113)^10^. Similarly to our VPS13C sample, the fungal Vps13 fragment used in the reconstruction, here referred to as *Ct*Vps13α, was purified from Expi293 cells, and we also found density corresponding to CaM in this reconstruction (Fig. 1D). The CaM binding site is equivalent in VPS13C and the fungal Vps13.

Figure S3 and Table 1 show representative portions of the maps and statistics for data collection, processing, and modeling.

### Architectural overview of the VPS13C-CaM complex.

At low resolution, VPS13C resembles a bubble wand, with the bridge-domain as a ~300 Å long handle and the C-shaped VAB domain as the loop. As predicted by AlphaFold, the bridge domain comprises N- and C-terminal “caps” at either end of the handle, with RBG motifs arrayed in between (Fig. 1 C, E). The VAB is an insertion between RBGs 12 and 13, and interacts with the bridge domain via linkers in its C-terminal “cap”. The WWE and PH domains are at the convex back face of the taco shell. CaM is bound at the N-terminal end of the bridge domain (Fig. 1C).

### Bridge domain.

Residues on the convex solvent exposed exterior of the bridge domain’s taco shell are hydrophilic, whereas residues lining the interior comprising the lipid transport path are hydrophobic (Fig. 1F). Lipid fatty acyl moieties are expected to bind within the hydrophobic channel, with their headgroups exposed to solvent. The channel appears filled at lower contour levels (Fig. S3A), but we could not resolve individual lipids. This is due to the limited resolution of the reconstructions and also because each reconstruction represents the average of hundreds of thousands of particles, where lipids may not have well defined binding sites. We estimate that the channel can accommodate ~135 lipids. The channel’s width varies along the length of the bridge domain: it measures ~20 Å across at its narrowest and ~50 Å across at its broadest points.

In addition to VPS13C and *Ct*Vps13α modeled by us here (Fig. 1D–E), several other experiment-based structures have recently become available for other BLTPs: VPS13A^11^, ATG2^14^, and an N-terminal fragment of BLTP-1^15^. The width of the lipid transfer channel is variable along the length of the bridge domain in all these proteins. Whether this variability is physiologically significant is unclear. Notably, however, the lipid transfer channel is wider in all VPS13 proteins compared to either ATG2 or BLTP1 (Fig. 1G–H), and it is significantly wider in VPS13C even as compared to VPS13A^11^ or fungal Vps13 (Fig. 1G–I). We posit that the width of the lipid transfer channel could impact lipid flux, so that the VPS13s, and VPS13C in particular, may be able to transfer lipids at faster rates than other narrower BLTPs. Lending plausibility, a recent manuscript reports that mutations that constrict ATG2’s width affect lipid transfer rates in cells^16^.

### CaM is a VPS13 interaction partner at the bridge domain’s N-terminal.

**Figure 2.**
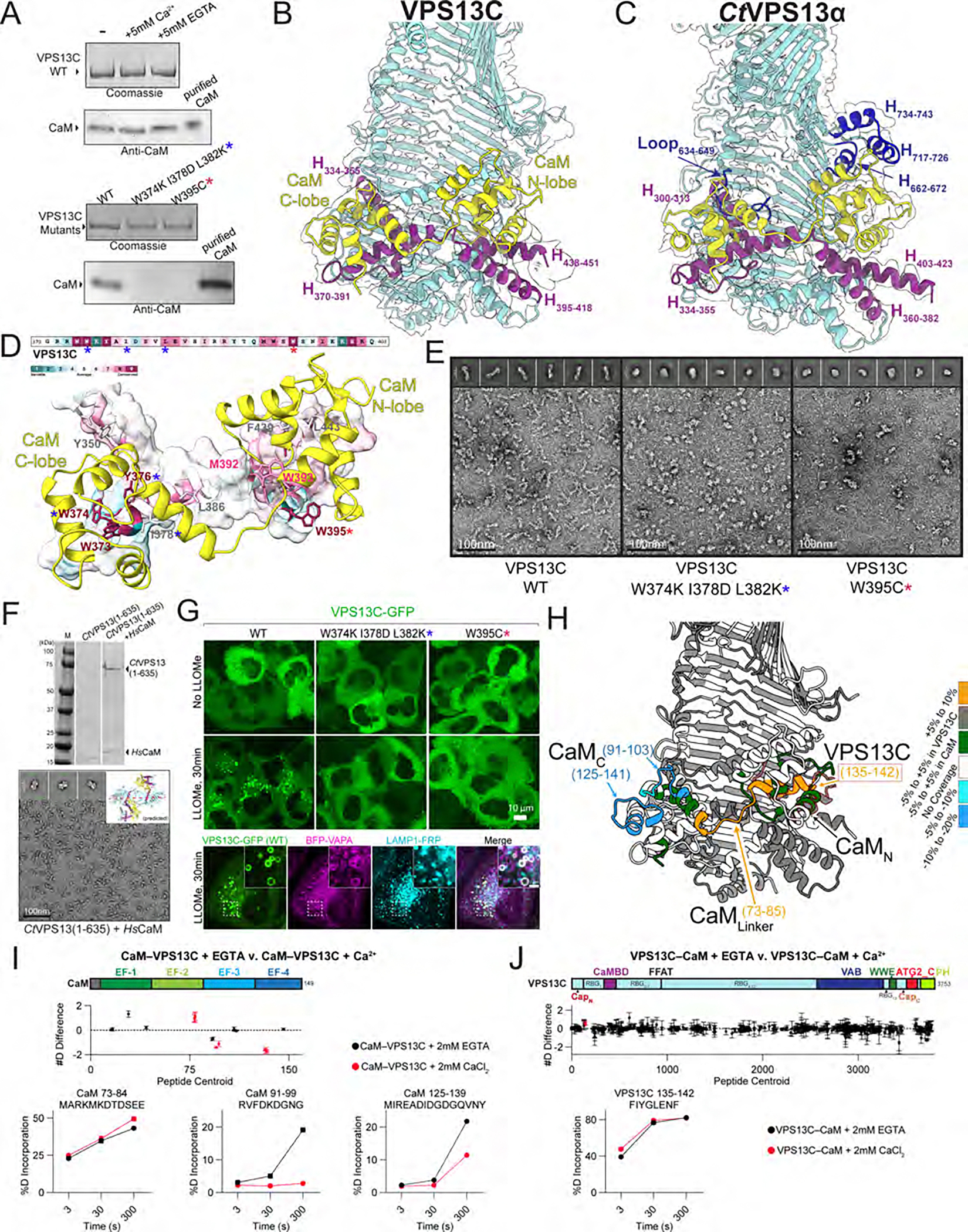
CaM constitutively binds VPS13’s N-terminus. (**A**) Co-purified CaM in FLAG-IP, detected by western blot. CaM association with VPS13C is unaffected by the presence of calcium versus EGTA (Top). The interaction is abolished by mutations in VPS13C at the interface with CaM’s N- or C-terminal lobes (Bottom). The white dashed line indicates where irrelevant lanes are digitally eliminated. (**B**) At VPS13C’s N-terminus, CaM’s N-terminal lobe interacts with H_395–418_ and H_438–451_, while the C-terminal lobe interacts with H_334–355_ and H_370–391_ between RBG_1_ and RBG_2_. (**C**) At *Ct*VPS13’s N-terminus, CaM’s N-terminal lobe interacts with H_360–382_ and H_403–423_, and its C-terminal lobe interacts with H_300–313_ and H_334–355_. Loop_634–649_, H_662–672_, H_717–726_, and H_734–743_ between RBG2 and RBG3 contact CaM near its EF-hand turns. (**D**) VPS13C is colored according to conservation score^35^. Conserved hydrophobic residues at VPS13C-CaM interface are shown as sticks. € Representative negative-stain EM images and 2D averages show that the W374K/I378D/L382K mutant and W395C mutants do not aggregate, but do not adopt WT VPS13C’s rod-like shape (see also Fig. S4). Scale bars, 100 nm. (**F**) In *E.coli*, *Ct*VPS13 (residues 1–635) requires CaM for soluble expression. The gap indicates where irrelevant lanes are digitally eliminated. Negative stain EM shows CtVPS13_1–635_-CaM dimerizing, consistent with AlphaFold3 predictions. Scale bars, 100 nm. (**G**) After LLOMe-induced lysosome damage in HeLa cells, wildtype VPS13C forms punctae at ER-lysosome contacts (bottom) while CaM-binding-deficient VPS13C mutants remain dispersed in the cytosol. Scale bars, 10 μm; inset, 2 μm. (**H**) Peptides in VPS13C-CaM that show significant HDX differences in the absence and presence of calcium (significant defined as >0.4 Da, >5% change, and p < 0.01 two-tailed t-test) are mapped onto the model of VPS13C’s N-terminus. Residues 120–148 of VPS13C (red outline), unresolved in the experimental structure, are modeled by AlphaFold; residues 135–142 are predicted as an α-helix (pLDDT 50–70) adjacent to the first EF-hand motif in CaM. (**I-J**) Top panels show the sum of the number of deuteron difference for all peptides across the full exchange time course for CaM (I) and VPS13C (J) in the presence of Ca^2+^ versus EGTA. Peptides showing significant HDX changes are in red. Each point represents an individual peptide; error bars indicate the summed standard deviation across all time points (n=3 per time point). Bottom panels display the mean deuterium exchange profiles (error bars show s.d., n=3) for select CaM or VPS13C peptides exhibiting significant changes between conditions. Also see Fig. S4.

Mass spectrometry analysis of proteins that co-immunoprecipitated with overexpressed VPS13C from Expi293F cell extracts identified CaM as a major hit (Table S2). We confirmed that CaM was present in the human VPS13C sample used in structural studies by western blotting (Fig. 2A). CaM is also reported as an interactor of VPS13A, C and D, but not VPS13B (the most divergent member of the VPS13 family), in proteomics databases (BioGRID) and was detected by western blotting of VPS13A purified from Expi293 cells.^11^ Moreover, an N-terminal fragment of VPS13D was identified in a screen for CaM interactors in *C.elegans*^17^. Consistent with our findings, AlphaFold^18^ predicts with high confidence a complex between CaM and human VPS13A, C, and D, but not VPS13B, and in these predictions CaM is positioned where the previously unexplained density in our maps of the VPS13C N-terminus was localized (map #3) (Fig. S4). Accordingly, we modeled CaM into this density (Fig. 2B). The interfaces between CaM and VPS13s are large (total buried surfaces between CaM and VPS13C or VPS13A, respectively, are 4380 A^2^ and 5713 A^2^; these numbers represent the sum of occluded surfaces in VPS13 and CaM), indicative of a high affinity interaction^19^. In the VPS13C reconstruction, CaM binds to four helices (residue ranges are indicated as subscripts: H_334–355_, H_370–391_, H_395–418_, and H_438–451_) that span across the bridge domain to connect the first and second RBG (Fig. 2B). CaM binds equivalent helical elements in another experimental structure, of VPS13A^11^. VPS13B, which does not appear to bind CaM based on proteomics databases (BioGRID), lacks the series of helices that form the primary CaM binding site (Fig. S4).

CaM comprises an N- and a C-terminal lobe, each of which can interact with substrate proteins. Each lobe has two EF-hands, which are helix-turn-helix motifs that bind calcium in the loop between helices^20^. In the structures of the VPS13-CaM complex, both lobes engage with VPS13. The N-terminal lobe interacts primarily with VPS13C’s H_395–418_, and the C-terminal lobe interacts primarily with VPS13C’s H_370–391_. VPS13 residues at the interface are conserved and hydrophobic, interacting with hydrophobic surface cavities on CaM (Fig. 2D).

We confirmed by western blotting that CaM also co-purifies with *Ct*Vps13α recombinantly produced in Expi293 cells (Fig. S4), and we found density corresponding to CaM in the map for *Ct*VPS13 (Fig. 1D, 2C). In a previous study^10^, we had described this density as part of a “handle” arching over the “basket”-shaped bridge domain and had incorrectly attributed it to Vps13. The interface area is even larger than that of the human VPS13s and CaM (6052 A^2^ total buried surface areas). As in the VPS13C and VPS13A cryo-EM structures, CaM interacts with helices that span between RBGs 1 and 2 (*Ct*H_300–313_, *Ct*H_334–355_, *Ct*H_360–382,_ *Ct*H_403–423_). Additionally, the calcium binding loop of the N-terminal lobe’s second EF-hand interfaces with a 3-helix bundle formed from loops of Vps13’s third RBG repeat (*Ct*H_662–672_, *Ct*H_717–726_, *Ct*H_734–743_). A similar interaction is present in VPS13A-CaM^11^, but not in VPS13C-CaM. Finally, the C-terminal lobe of CaM interacts with a loop in *Ct*Vps13’s second RBG repeat (residues 634–649). Note that endogenous yeast Vps13 was found to co-purify with Cdc31, a member of the CaM superfamily^21,22^. Based on AlphaFold prediction, Cdc31 binds where CaM is bound in the cryo-EM reconstruction of recombinant *Ct*Vps13 (Fig. S4). We note that other members of the CaM superfamily, namely S100 proteins (Table S2), also co-immunoprecipitated with VPS13C, although not with the same enrichment as CaM. Thus, the interaction between VPS13s (except VPS13B) and proteins in the CaM superfamily is conserved across eukaryotes.

To further confirm the importance of the helical segment between VPS13 RBG repeat 1 and RBG repeat 2 for CaM binding, we introduced mutations in this region of VPS13C, expecting to abrogate its interaction with CaM. We mutated residues along H_370–391_ or H_395–418_ to disrupt binding to CaM’s C-terminal or N-terminal lobe (Fig. 2A, D, Fig. S4), respectively. W395C and A444P are of particular interest because two siblings compound heterozygous with these missense mutations were affected by Lewy-Body dementia, a disease closely related to Parkinson’s^23^. All our mutant constructs were expressed at levels similar to the wild-type protein in our Expi293 overexpression system and did not form aggregates, such as would result from severe misfolding, as assessed by size exclusion chromatography (i.e., they were not in the void volume) or negative stain EM (Fig. 2A, E, Fig. S4G–K). Consistent with a role for the helical segment linking RBG 1 and RBG 2 in binding CaM, the mutants did not co-purify with CaM (Fig. 2A, Fig. S4H). Interestingly, though, the mutant proteins had different profiles by size exclusion chromatography indicative of different hydrodynamic radii, and they did not form rods like wild-type protein as determined by negative stain microscopy (Fig. 2E, Fig. S4G, I).

One explanation for these data is that CaM is a constitutive binding partner for VPS13 and the mutants misfolded because they could not bind CaM, thus exposing to solvent hydrophobic VPS13 residues normally be buried in the interface. As CaM is present and highly expressed in the Expi293 cells we used for protein production, we turned to a bacterial expression system, where CaM is not present, to assess whether wild-type VPS13 constructs require CaM for folding. We worked with a 70kDa fragment of *Ct*Vps13 (residues 1–635), small enough for bacterial expression, which includes the tandem helices between RBG 1 and RBG 2 that comprise the primary CaM binding site. While *Ct*Vps13_1–635_ by itself was poorly expressed and not soluble, we were able to co-express it with CaM, obtaining good yields of a *Ct*Vps13_1–635_-CaM complex (Fig. 2F). *Ct*Vps13_1–635_ and CaM assemble into a heterotetramer, with the two copies of CtVps13_1–635_ arranged tail-to-tail into a rod. This tail-to-tail arrangement was also observed in previous experimental structures of *Ct*Vps13 fragments^5,10^, and is predicted by AlphaFold for *Ct*Vps13_1–635_ (Fig. 2F). Thus, this experiment supports the notion that CaM, or perhaps another member of the calmodulin superfamily, is constitutively associated with CtVPS13 and is required for CtVPS13 to fold into a rod-like shape. As the CaM binding site is well conserved across VPS13 family members (except VPS13B) (Fig. 2D, Fig. S4), the experiment also strongly implies that CaM proteins are constitutive binding partners for other VPS13s (except VPS13B), so that misfolding would be expected for any mutant unable to bind CaM.

The location of CaM’s binding site near the N-terminal end of VPS13C, where it interacts with the donor organelle, i.e. the ER^5^, raised the possibility of a role for CaM in VPS13 localization. Under control conditions much of VPS13C is soluble in the cytosol, with only a pool of VPS13C (whose proportion varies in different cell types) at lysosomes^5,9^. However, damage of the lysosome membrane, for example in response to addition of the lysosomotropic compound LLOMe^9^, induces a massive redistribution of WT VPS13C to these organelles with the formation of ER-lysosome contacts (Fig. 2G). As we had previously shown, pre-treating cells with the calcium chelator BAPTA-AM prior to addition of LLOMe did not alter VPS13C recruitment in response to this compound^9^. Thus, cytosolic calcium is not required for LLOMe-mediated recruitment of VPS13C to ER-lysosome contacts. However, the association of VPS13C with CaM is required, as we found that CaM-binding deficient mutants, including Lewy-Body mutants W395C and A444P, remain in the cytosol and fail to be recruited to either the lysosome or the ER (Fig. 2G, Fig. S4G) (and^23^). The data do not allow us to distinguish whether the failure to localize correctly is because CaM is required for a direct association with the ER, or else because it is required for the correct folding of the VPS13C N-terminal end, so that this can interact directly with the ER.

We also tested whether elevation of cytosolic calcium had an impact on the localization of VPS13C. To this aim we used thapsigargin (1 μM)^24^, which, as expected, produced a robust transient increase in the fluorescence of calcium sensor r-GECO in the RPE1 cells used for this experiment (Fig. S5A). We found no effect on the localization of VPS13C, which in these cells was already partially localized at lysosomes before any manipulation (Fig. S5A and Video SV1), although in control experiments we showed that addition of LLOMe to the same cells under the same conditions triggered robust additional VPS13C recruitment to ER-lysosome contacts (Fig. S5B and. Video SV2). These findings were confirmed by biochemical experiments showing that treatment of HEK293T cells expressing FLAG-VAP with 1 μM thapsigargin for 1 min (when cytosolic calcium elevation peaked, as recorded by r-GECO) did not affect the pool of endogenous VPS13C that copurified by FLAG-GFP (Fig. S5C–E and Video SV3). We also tested the effect of thapsigargin in RPE1 cells expressing a N-terminal CaM binding fragment of VPS13C (residues 1–1390), which contains the FFAT motif for ER binding, and found no effect on the localization of this construct, which is primarily cytosolic (Fig. S5F and Video SV4). In conclusion, these experiments indicate that calcium dynamics alone are not responsible for VPS13C localization. They do not exclude, however, that calcium dynamics could contribute to localization of VPS13C under certain functional states or that calcium may affect its lipid transfer function, either via direct actions on the VPS13C-CaM complex or indirectly, for example via post-translational modifications.

To probe whether calcium might induce conformational changes in the VPS13C-CaM complex, we carried out hydrogen-deuterium exchange mass spectrometry (HDX-MS) experiments on the complex in the presence (2 mM Ca^2+^) or absence (2 mM EGTA) of calcium. HDX-MS measures the exchange rate of protein amide hydrogens with deuterated solvent, allowing for assessment of solvent accessibility and modification of such accessibility as a result of conformational changes. We used a truncated version of VPS13C lacking the amphipathic helices in the ATG2_C motif (VPS13ΔATG2_C), which are at the C-terminal end of VPS13C far from the CaM binding site. This was required to obtain sufficient material to carry out a full HDX-MS experiment. We were able to obtain peptides covering the majority of both VPS13C (69.7%) and CaM (66.4%). Full experimental details are in the source data. For CaM, there were significant decreases in exchange with calcium in both EF-hands of the C-terminal lobe, and significant increases in exchange in the linker region between the lobes (Fig. 2H–J, Fig. S4L–N). We also observed calcium-responsive changes in VPS13C, with increased exchange in an unstructured loop near the N-terminus (peptide spanning residues 135–142). Thus, the HDX-MS data support that calcium can induce conformational changes in the VPS13C-CaM complex.

### Interactions with the C-terminal “cap” stabilize the VAB in a lipid transfer inactive conformation.

**Figure 3.**
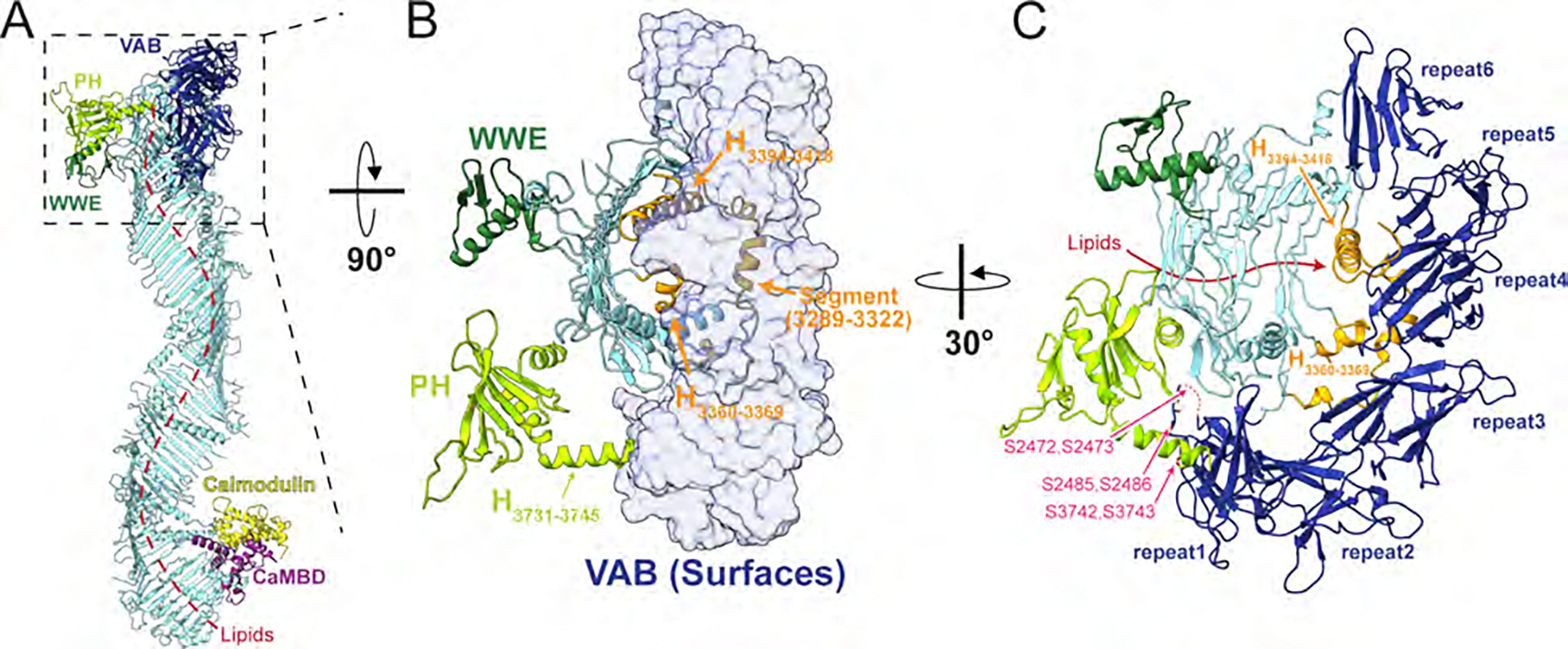
VPS13C adopts a lipid transfer-incompetent conformation at its C-terminus. (**A**) Side view of the VPS13C showing the VAB domain (dark blue) covering the lipid transfer channel’s C-terminal end, preventing membrane docking and lipid transfer. The red dotted line indicates the presumed lipid transfer path. (**B**) VPS13C’s C-terminal end as viewed from the lysosome membrane. The VAB domain (pale blue surface) makes extensive contacts with the C-terminal cap (orange) through H_3418–3394_, H_3360–3369_, and an extended segment (3289–3322). (**C**) Loops from the C-terminal cap bind to the VAB’s beta-sandwich repeats #3 and #4. H_3418–3394_, H_3360–3369_, and the VAB domain obstruct the lipid transfer path (red arrow). Additionally, H_3731–3745_ from the PH domain contacts beta-sandwich repeat #1. Phosphorylation of residues in the VAB’s first beta-sandwich repeat (S2485, S2486, S2472, S2473) and C-terminal residues from the PH domain in H_3731–3745_ (S3742, S3743), in pink, may contribute to conformational changes in the VAB domain allowing the bridge domain to access membrane.

In the cryo-EM reconstruction of VPS13C we see that the six beta-sandwich repeats that comprise the VAB domain arch over the end of the bridge domain, positioned via extensive interactions with the C-terminal “cap” (3507 Å^2^ occluded surfaces)(Fig. 1E, 3A–C). This “cap” (in orange in Fig. 1E) comprises two beta strands and an alpha helix (H_3394–3418_) that lies across the bridge-domain, partially closing off the lipid transfer channel. The segment connecting RBG 13 and the “cap” (residues 3289–3322) snakes along the concave surface of the C-shaped VAB, contacting beta-sandwich repeats #2-#5, and the connecting loop between the “cap”’s two beta strands contacts sandwich repeat #4 (Fig. 3C). Additionally, in VPS13C, the end of the helix C-terminal to the PH domain butts against beta-sandwich repeat #1 (Fig. 3B–C). For lipid transfer to occur, both the VAB and H_3394–3418_ need move away, allowing the bridge domain access to membrane and unblocking the transfer channel.

In the cryo-EM study of another VPS13, VPS13A, in complex with a receptor at the acceptor membrane XK^11^, the VAB has moved to the side of the bridge domain, and segments of the “cap” that interact with the VAB in the VPS13C structure are not visible in the maps, presumably because they are mobile. Nor is there an alpha helix corresponding to VPS13C’s H_3394–3418_ blocking the end of the lipid transfer channel. Our VPS13C model here represents a lipid-transfer nonpermissive conformation, but very likely VPS13C can undergo conformational changes to a lipid-transfer active conformation represented in the VPS13A structure reported by Hu et al.^11^

### VPS13C recruitment to damaged lysosomes.

Previous *in cellulo* studies showed that (i) VPS13C is localized at contacts between the ER and lysosomes^5^ (Fig. 2G), (ii) that this recruitment depends on VPS13’s VAB domain and the lysosomal small GTPase Rab7^9,25^ and (iii) that recruitment is strikingly enhanced by lysosome damage.^9^ It was not known, however, whether VPS13C and Rab7 interact directly, or how.

**Figure 4.**
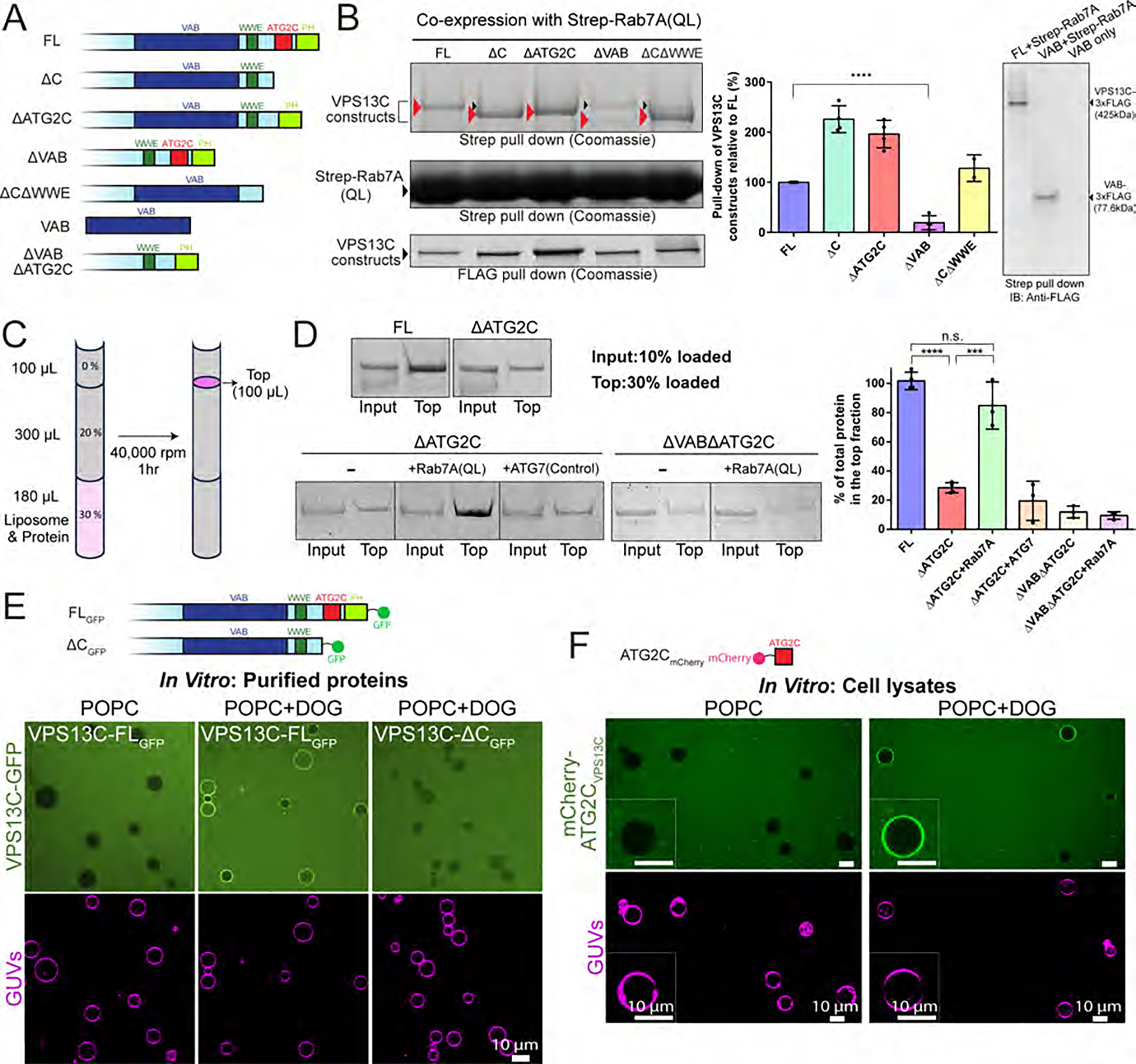
VPS13C membrane recruitment. (**A**) VPS13C constructs used for binding assays. (**B**) Left and middle panels show that Strep-tagged Rab7A (Q67L) pulls down full length (FL) FLAG-tagged VPS13C when co-expressed in cells, and this interaction requires the VAB but not the PH domain, WWE domain, or ATG2_C motif. Only VAB domain deletion drastically reduces co-purification. Red arrowheads indicate FLAG-VPS13C constructs; black arrowheads represent endogenous VPS13C. Anti-FLAG Ips of the starting lysate (bottom of left panel) shows expression levels of the constructs. Quantification of VPS13C constructs retained by Rab7 is in the middle panel. The band intensity of each VPS13C construct retained by Rab7 is normalized by the corresponding Rab7 band intensity, and then normalized to the full-length (FL) construct. N=4 biological replicates, except ΔC+WWE, where n=2. Data were shown as mean ± s.d. and compared using a two-sided Student’s t-test; ****P<0.0001. The right panel shows retention by Rab7A (Q67L) of both FL VPS13C or its VAB, detected by western blot. The white dashed line indicates where irrelevant lanes are digitally eliminated. (**C**) Schematic of the liposome floatation assay. VPS13C co-flotation with small unilamellar vesicles (SUVs) correlates with its membrane binding ability. (**D**) ~100% of VPS13C-FL and ~30% of VPS13C-ΔATG2C co-float with SUVs (top). Recruitment of VPS13C-ΔATG2C is restored (>80% co-floatation) by tethering His-tagged Rab7A, but not a negative control (His-tagged ATG7), to SUVs. Flotation of the VPS13C-ΔVABΔATG2C construct was not rescued by Rab7A tethering. The graph quantifies the fraction of VPS13C constructs that co-floated with SUVs; n=4 for FL and ΔATG2C alone; n=3 for others, where n represents biological replicates. Data shown as mean ± s.d. and compared using a two-sided Student’s t-test; ****P<0.0001; ***P<0.001; n.s., not significant. (**E**) Purified GFP-tagged VPS13C-FL only localizes to giant unilamellar vesicles (GUVs) composed in part of dioleoylglycerol (DOG) lipids, which induce packing defects (Fig. S6). In contrast, VPS13C-ΔC does not localize to GUVs, even in the presence of DOG lipids. Scale bars, 10 μm. (**F**) mCherry-tagged ATG2_C motif binds to GUVs with DOG lipids (see methods). Scale bars, 10 μm.

We undertook protein-protein interaction experiments to obtain a better understanding of how VPS13C and Rab7 might bind each other (Fig. 4). In these experiments, we co-expressed strep-tagged Rab7 (to ensure the GTP-bound “active” form present at the lysosome, we used a constitutively active mutant, Rab7(Q67L)) and FLAG-tagged VPS13C or VPS13C deletion mutants (Fig. 4A), passed cell lysates over Strep-Tactin resin to retain Rab7(Q67L) and associated proteins, washed the resin to remove non-specifically bound proteins, and finally eluted Rab7(Q67L) and associated proteins. The eluted proteins were analyzed by SDS-PAGE and visualized by Coomassie stain and/or western blotting. Expression of Flag-tagged VPS13C constructs in the original lysates was assessed by anti-FLAG immunoprecipitation (Fig. 4B).

We found that Rab7(Q67L) specifically retains full-length VPS13C, although with relatively weak affinity as the retained VPS13C represented only a small fraction (~5%) of total overexpressed VPS13. The interaction depends on the presence of VPS13C’s VAB domain since retention of a VPS13C construct lacking the VAB was drastically reduced. Removing other C-terminal modules—including the amphipathic helices in the ATG2_C motif or the WWE or PH domains—or combinations of them did not abrogate VPS13C pull-down, indicating that these regions are not required for interactions with Rab7. A construct encoding the VAB domain alone was retained by Rab7, demonstrating sufficiency of the VAB domain for Rab7 binding (Fig. 4B). We were also able to isolate a complex between Rab7 and VPS13C using purified proteins, in the presence of cross-linker as the interaction is weak (Fig. S1F), supporting that the interaction is direct (but see below).

Amphipathic helices are well known to insert into lipid mono- and bilayers^26^, particularly those with packing defects as might be found in damaged lysosomes^27^. Moreover, previous *in cellulo* studies suggested that amphipathic helices in VPS13’s ATG2_C motif act to sense lysosome damage and bind to their membranes.^9^ To complement these earlier studies, we carried out liposome-based assays with purified proteins. In agreement with the cellular studies, we found that full length VPS13C associated with small unilamellar vesicles (SUVs) in flotation assays (Fig. 4 C–D), whereas the association of a VPS13C construct lacking the amphipathic helices of the ATG2_C motif, VPS13CΔATG2C, was strongly reduced (~70% reduction). Binding was not entirely eliminated, likely because other elements at both the N- and C-termini of VPS13CΔATG2C also interact with membranes. VPS13CΔATG2C’s association with SUVs was rescued by tethering purified Rab7(Q67L) to the SUVs via a hexahistidine tag bound to DGS-NTA lipids (Fig. 4D). The constitutively “inactive” GDP-bound mutant of Rab7 (T22N) did not rescue VPS13C ΔATG2C flotation, indicating that VPS13 is sensitive to the nucleotide-bound form of the Rab (Fig. S6). Further, consistent with a role for the VAB domain in interactions with Rab7, the presence of Rab7(Q67L) did not rescue liposome association of another VPS13C construct that additionally lacked the VAB domain (VPS13CΔVABΔATG2C) (Fig. 4D). Importantly, as these experiments were carried out with purified proteins rather than with cell lysates, they provided strong support that the interaction between Rab7 and VPS13C is direct, involves the VAB domain, requires the GTP-bound form of Rab7, and occurs in the context of membrane.

In a separate experiment, we monitored the association of purified fluorescently tagged constructs of VPS13C with giant unilamellar vesicles (GUVs) by confocal microscopy. Full-length VPS13C did not associate with POPC-GUVs, but only with POPC+DOG-GUVs (containing 30% dioleoylglycerol (DOG)), where DOG introduces packing defects due to its smaller head group (Fig. S6C–E)^28^. Further, even binding to GUVs containing DOG was no longer observed when a C-terminally truncated VPS13C lacking the ATG2_C motif and PH domain (DC_GFP_) was used (Fig. 4E), supporting that the ATG2_C motif is required for membrane binding. We next demonstrated sufficiency of the ATG2_C motif for membrane binding. As the ATG2_C fragment of VPS13C could not be purified due to aggregation, we expressed it as an mCherry-tagged construct in Expi293 cells and incubated lysates from these cells with GUVs, finding that colocalization of the protein with GUVs occurs only with GUVs containing DOG (Fig. 4F). Th experiments showed that VPS13C can sense the difference between membranes with and without packing defects, supporting the importance of lysosome damage in the recruitment of VPS13C. Note that full-length VPS13C behaves differently in the GUV-based assays (Fig. 4E) versus in the flotation assay, where binding does not require DOG (Fig. 4C–D). This may be due to the high curvature of the liposomes used for the flotation assay, as high curvature will decrease the packing of phospholipid head groups, or to the different lipid compositions of the liposomes used for the two assays.

Collectively, our in vitro experiments agree with and expand upon the conclusions from experiments in cells, that VPS13C localizes to damaged lysosomes via coincidence detection: the amphipathic helices sense membranes with packing defects, and the VAB domain interacts with Rab7 on lysosomal membranes.

## Discussion

**Figure 5.**
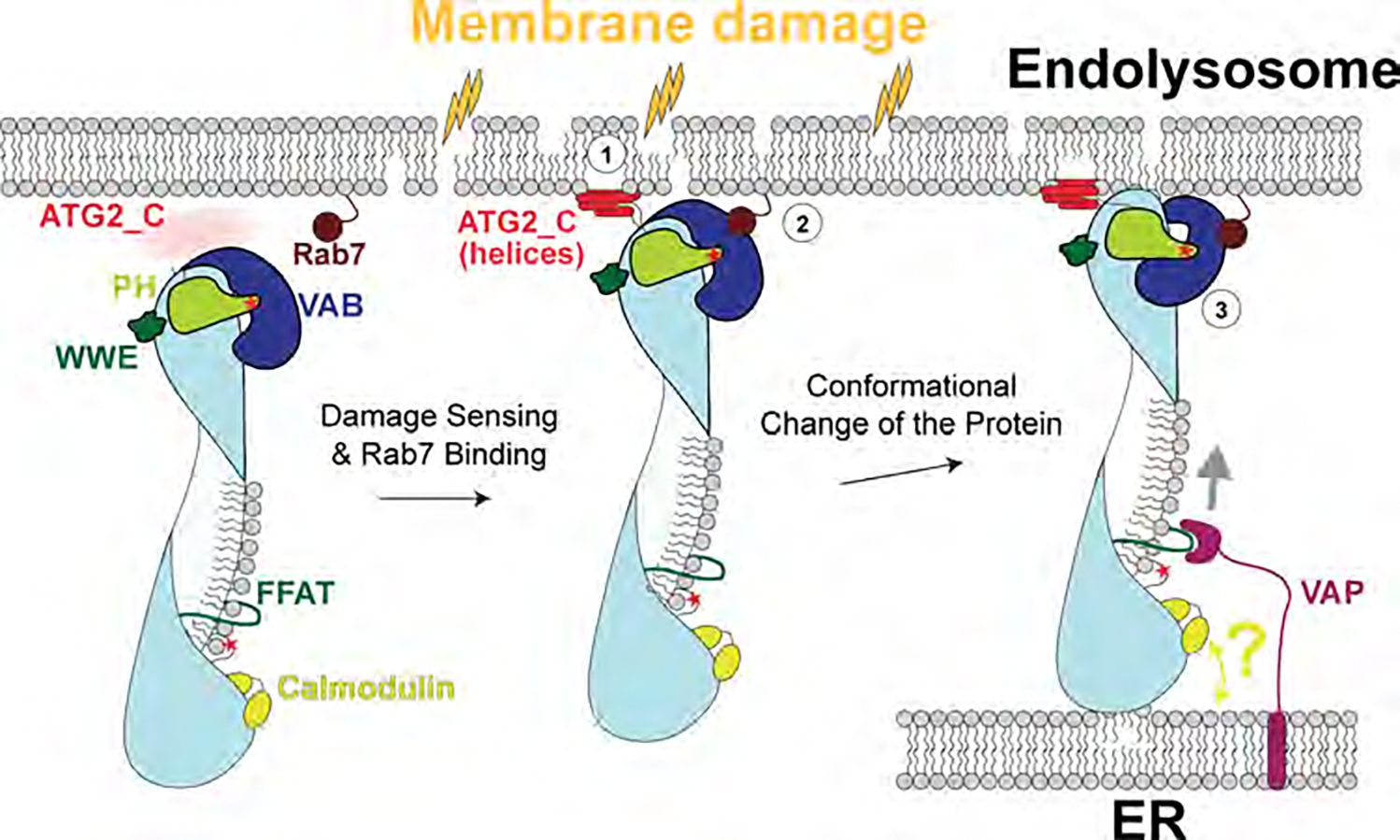
Mechanisms for VPS13C regulation. At the C-terminus, the VAB covers the end of the bridge domain, where lipids would be transferred to membrane, blocking lipid transfer. Upon lysosomal membrane damage, amphipathic helices in the ATG2_C motif (mobile in the soluble autoinhibited state, indicated by “fuzzy” red color) associate with the membrane, and the Rab7A-VAB interaction further stabilizes VPS13C’s membrane attachment. Conformational changes in the VAB domain expose the lipid bridge for transfer. At the N-terminus, CaM binds constitutively and may modulate bridge architecture and/or ER recruitment in response to calcium. Phosphorylation at both termini (red stars) by kinases in calcium signaling pathways may further regulate VPS13C conformation and its activity at contact sites.

Formation and molecular composition of membrane contacts within cells are dynamically regulated in response to cellular signals. It is increasingly appreciated that BLTPs are key players at these sites via their lipid transfer function, but how their localization, assembly into complexes, and function is controlled is still far from understood. Our work on one such protein, VPS13C, has uncovered new aspects of its regulation, and of VPS13 family protein regulation more broadly (Fig. 5).

Previous studies in cells had suggested that VPS13C might exist in different conformations, as this would explain why a construct encoding only its VAB domain binds lysosomes constitutively, while binding to these organelles by full-length VPS13C is highly regulated^9^. More specifically, it was shown that VPS13C is present in the cytosol in an autoinhibited state from which it is released when the C-terminal region of VPS13C interacts with damaged lysosomal membranes. One major outcome of our cryo-EM study was to identify an inactive conformation for VPS13C which likely corresponds to this autoinhibited state. We found that when VPS13C is in solution, interactions with the C-terminal “cap” maintain the arch-shaped VAB domain over the end of the bridge domain. In this position, the VAB blocks access of the bridge domain to the acceptor bilayer, thus preventing lipid transfer. We also identified “cap” helix H_3394–3418_ as partially blocking the end of the lipid transfer channel in this lipid-transfer nonpermissive state. Thus, lipid transfer requires conformational rearrangements at the VPS13 C-terminal end, where “cap” helix H_3394–3418_ moves out of the lipid transfer channel and interactions between the “cap” and VAB are loosened, allowing the VAB to move to the side of the bridge and thus to remove the occlusion of its end. Indeed, in a recent cryo-EM study of VPS13A complexed with a receptor (the scramblase XK) at the acceptor membrane^11^, VPS13A is in such a transfer permissive conformation. It is likely that the existence of lipid-transfer inactive and active conformations is a feature of all VPS13 proteins as they mostly share the same domain architecture, and thus likely share regulatory mechanisms.

Our present and past^9^ results suggest that the coincident insertion of the amphipathic helices of the ATG_C motif into the lipid packing defective membrane of the damaged lysosome and association of the VAB domain with Rab7 could help trigger the conformational transition between lipid transfer inactive and active forms for VPS13C. In a previously proposed model, access of the VAB domain to Rab7 is prevented by VPS13C’s C-terminal modules while VPS13C is cytosolic^9^, but the validity of this model remained to be assessed by biochemical and structural studies. Our work here reveals that conformational changes are required not only for Rab7 to access its binding site on the VAB domain but also to shift VPS13 from a lipid-transport nonpermissive to a lipid-transport permissive state (as in Fig. 5). There likely are additional triggers for the conformational changes that are not yet identified. These could include changes induced by elevation of cellular calcium levels and by post-translational changes as discussed further below, or by still undiscovered interaction partners.

A second major outcome of our study is identification of calmodulin as an interactor for the subset of human VPS13s (VPS13A, VPS13C, VPS13D, but not VPS13B) that localize to contacts involving the ER as the lipid donor membrane. We found that CaM also interacts at an analogous site with fungal Vps13, although the predominant physiological binding partner of yeast Vps13 may be another member of the calmodulin superfamily, Cdc31^21,22^, rather than CaM itself. Thus, the function of calmodulin proteins in VPS13-mediated lipid transport is conserved across species, from yeasts to mammals. The binding site for CaM in VPS13 is close to the N-terminal end of its bridge domain, proximal to the long “FFAT”-motif loop (residues 877–883 in VPS13C) that mediates binding of VPS13 to the ER protein VAP.^5^ As the association of the N-terminus of the bridge domain of VPS13 to the ER is required to allow VPS13 to function in lipid extraction from this organelle, an attractive model is that CaM helps control the connection between the bridge-domain and the ER membrane. So far, though, we have no evidence for such a role. As the HDX-MS experiments indicate that calcium induces conformational changes within the VPS13C-CaM complex at sites where the two proteins interact, calcium-induced conformational changes could be involved in activating the complex for lipid transfer or controlling lipid flux through it.

The presence of CaM, which is central to intracellular calcium signaling, in VPS13 complexes converges with other findings implicating VPS13 activity in calcium dependent pathways, either directly or indirectly^29,30^. In particular, Soczewka et al.^29^ reported that sequestration of CaM could ameliorate a phenotype due to VPS13 deficiency in yeast. Since this rescue occurred in the absence of VPS13, the effect must be indirect, likely due to decreased calcineurin activity, as reported in a follow-up study^31^. A link between calcium and VPS13 is especially appealing because intracellular membrane contact sites are hubs for calcium signaling.

Many calcium signals are transmitted via post-translational phosphorylation, often involving calcium/calmodulin-dependent kinases (CaMKs). In this context, it is noteworthy that inspection of phosphosite databases reports several sites expected to be controlled by calcium/CaM-dependent kinases (Phosphosite.org), especially at the N- and C-terminal ends of VPS13C likely to interact with organellar membranes. We noted before that in the reconstruction, the end of H_3731–3745_ at the very C-terminus of VPS13C butts against a hydrophobic patch on beta-sandwich repeat #1 of the VAB, and we find intriguing that residues at the end of the helix (S3742, S3743) and in a loop of VAB domain’s repeat #1 (S2473, S2485) near the bridge domain are reported to undergo phosphorylation (Phosphosite.org). Plausibly their phosphorylation could help precipitate the VABs rearrangement to a lipid-transfer competent conformation. Further, residue S373 in a loop near the CaM binding site and residues of the FFAT motif also are reported to be phosphorylated (Phosphosite.org). Phosphorylation of the FFAT motif may enhance VPS13C’s recruitment by VAP on the ER^32^; and, indeed, VPS13D features a 10arin10or-FFAT motif, whose phosphorylation is required for its ER-localization^33^. How post-translational modifications impact VPS13 activity is a fertile ground for future investigation, albeit beyond the scope of the current work.

In conclusion, as exemplified here for VPS13C, the localization of VPS13s and their lipid transfer activity at contact sites involves regulated interaction with protein partners at the interface with membranes; direct interactions with bilayers; regulation by calcium binding or post-translational modifications; and conformational rearrangements (Fig. 5). In sum, VPS13 activity depends on a highly complex interplay of multiple factors. Our work represents both a significant advance in our understanding of how VPS13s are activated and, moreover, a framework for further research regarding the function of this fascinating class of proteins.

### Limitations of the Study:

Although this study and a concomitant study on VPS13A^11^ show VPS13 family members in lipid-transfer competent and incompetent conformations, we do not yet know how the transition is triggered. Factors involved may include (i) membrane association of the amphipathic helices in the ATG2_C motif; (ii) the interaction of the VAB domain with Rab7 or the interaction of other C-terminal modules with still to be identified protein partners; (iii) changes in the membrane lipid composition at the acceptor membrane; (iv) signaling events dependent on cytosolic calcium or calcium regulated post-translational modifications or other signaling pathways; (v) allosteric changes along the VPS13 bridge domain. Most likely, the transition will be triggered by a combination of factors.

A major limitation in dissecting how these various factors contribute to VPS13 protein function and regulation is that we currently lack a physiologically relevant lipid transfer assay, either in cells or in vitro. In cells, we can assess VPS13C localization, but there is no assay to directly monitor lipid flow via VPS13C. In vitro, VPS13 and other BLTPs have been shown to transfer lipids between liposomes^5,34^, but in these assays the BLTPs can and most likely act as lipid shuttles rather than bridges, since the tethers and interaction partners that connect them with membranes are only incompletely identified. Moreover, there is as yet no *in vitro* assay featuring a driving force to promote directional lipid transfer between membranes, versus lipid equilibration. As the field develops such assays, we will be able to systematically assess contributions of different factors to function.

## Resource Availability

### Lead contact:

Further information and requests for reagents and resources should be directed to the lead contact, Karin Reinisch (11arin.reinisch@yale.edu).

### Materials availability:

Materials generated in this study will be shared by the lead contact upon request. Plasmids generated in this study were deposited to Addgene (see Table S1 for plasmid names and catalog numbers).

### Data and Code Availability:

Cryo-EM maps for VPS13C are available at the EMDB with accession numbers: EMD-73345, EMD-73344, EMD-73343, EMD-73373. Structural models have been deposited at the PDB with accession numbers: 9YRP, 9YQP, 9YQQ, 9YRM. HDX-MS data have been deposited in the PRIDE database with accession number PXD076522. Key lab materials, codes, datasets, and protocols used in this study are listed in a Key Resource Table alongside their persistent identifiers in Zenodo (doi.org/10.5281/zenodo.17459272). All source data are deposited in Zenodo (doi.org/10.5281/zenodo.17417584) and Mendeley (doi.org/10.17632/xgts777pxc.1). The customized script for Laurdan-GP analysis is deposited in Zenodo (doi.org/10.5281/zenodo.19688024); all data cleaning, preprocessing, analysis, and visualization was performed using Python, Microsoft Excel, and GraphPad Prism. Any additional information required to reanalyze the data reported in this paper is available from the lead contact upon request. An earlier version of this manuscript was posted to bioRxiv on 2025.10.11 at doi.org/10.1101/2025.11.10.687702.

## STAR Methods

### Experimental Models and Study Participant Details

For biochemical and Cryo-EM studies, Expi293F cells (RRID:CVCL_D615) were cultured at 37 °C with 8% CO_2_ in Expi293 expression medium (Cat. #A1435101, Gibco). Cells were cultured with constant orbital shaking at 125 rpm, following the manufacturer’s instructions (MAN0007814, Thermo Scientific). For bacterial protein expression, BL21(DE3) pLys cells (Cat. #200132, Agilent) were transformed and grown at 37°C in LB broth (Cat. #L24340, RPI) with shaking at 180 rpm.

For cellular experiments, HeLa cells (Cat. #RCB5388; RRID: CVCL_R965) were cultured at 37 °C with 5% CO_2_ in DMEM medium (Cat. #11965092, Gibco) supplemented with 10% FBS (Cat. #A5256701, Gibco). RPE-1 cells (Cat. #CRL-4000; RRID: CVCL_4388) were cultured at 37 °C with 5% CO_2_ in DMEM/F12 medium supplemented with 10% FBS and GlutaMAX (Cat. #3505006, Gibco). HEK293T cells (Cat. #CRL-3216, RRID:CVCL_0063) were cultured at 37°C with 5% CO_2_ in DMEM medium supplemented with 10% FBS, GlutaMAX, non-essential amino acids, and sodium pyruvate (Cat. #11360070, Gibco).

### Method Details

#### DNA Plasmids

All plasmids used in this paper are listed with corresponding RRIDs in Table S1 and deposited into Addgene. The codon-optimized full-length human VPS13C (UniProt: Q709C8) with a C-terminal 3xFLAG tag, human Rab7A-Q67L (UniProt: P51149) with an N-terminal Strep tag or an N-terminal MBP tag and a C-terminal 6xHis tag, Chaetomium VPS13 (UniProt: G0S3B8) with an N-terminal 3xFLAG tag were cloned into the pCAG vector. VPS13C mutants, ΔATG2C, ΔC, ΔVAB, ΔC+WWE and VAB domain were cloned into the pCAG vector with a C-terminal 3xFLAG tag. Other VPS13C constructs, including FL_GFP_, ΔC_GFP_, and all calmodulin-binding defective mutants were cloned into the pCMV10 vector with a C-terminal GFP tag, followed by 3xFLAG tag. All cloned plasmids were amplified using maxiprep (Catalog # 740414, Takara Bio) before transfection. For bacterial expression, the *Ct*VPS13 fragment (1–635), human Rab7A-Q67L, and Rab7A-T22N were cloned into the first multiple cloning site of the pETDuet vector with an N-terminal 6xHis-Strep tag. For co-expression of calmodulin, to the second multiple cloning site in pETDuet, the full-length human calmodulin sequence (*Hs*CaM, UniProt: P0DP23) was inserted with an N-terminal 6xHis tag.

#### Expression and purification of full-length VPS13C and its mutants

For transfection, 200 μg constructs encoding full-length VPS13C or its mutants were transfected using Expifectamine (Cat. #A14525, Gibco) into 200mL Expi293F cells at a density of 2.4–2.8 million cells/ml, and manufacturer supplied enhancers were added 18 hours post-transfection. Cells were harvested after 48 hours of transfection, flash-frozen and stored at −80°C until use. For protein purification, cells were thawed at room temperature and resuspended in protein purification buffer A (500 mM NaCl, 50 mM HEPES, pH 7.8, 10% glycerol, 1 mM TCEP), supplemented with 1x protease inhibitor cocktail (Cat. #11873580001, Roche). Cells were lysed by three rounds of freeze-and-thaw cycles between liquid nitrogen and a water bath at room temperature. The lysate was homogenized in a Dounce homogenizer and then centrifuged at 27,143 rcf for 30 minutes in a JA-20 rotor. The supernatant was incubated with 100 μL anti-FLAG M2 resin (Cat. #A2220, Millipore-Sigma) for 2 hours at 4°C. The resin was washed twice with 15 mL of buffer A, then incubated overnight with buffer A supplemented with 1 mM freshly-made ATP and 2 mM MgCl_2_ to remove chaperones. After two additional washes, proteins were eluted using Buffer A containing 0.25 mg/mL FLAG peptide (Cat. #A6002, Apex Bio). Elution was performed in five sequential 100 μL incubations, each lasting 20–30 minutes. The pooled eluates were loaded onto the Superose 6 10/300 column (Cat. #29091596, Cytiva), pre-equilibrated with buffer B (200 mM NaCl, 50 mM HEPES, pH 7.2, 6% glycerol, 1 mM TCEP). For structural analysis, full-length VPS13C was subjected to size-exclusion chromatography in buffer C (200 mM NaCl, 50mM HEPES, pH 7.2, 1mM TCEP). Peak fractions were collected and concentrated. The detailed protocol for expression and purification of VPS13C and related constructs were deposited in protocol.io (doi.org/10.17504/protocols.io.rm7vz92d4gx1/v1).

#### Negative stain electron microscopy of full-length VPS13C and its mutants

Negative stain analysis was performed using 400-mesh carbon-coated copper grids (Cat. #CF400-Cu, Electron Microscopy Sciences). Grids were glow-discharged for 35 s at 25 mA in a Sputter Coater (Cat. #SCD005, Bal-Tec). 5–7 μL of the protein sample, diluted to 50–100 nM, was applied to the grid and incubated for 30 seconds. The sample was blotted and three to five drops of 5 μL of 2% uranyl acetate solution were applied sequentially, with the last drop incubated for 30 seconds before blotting to complete dryness. Images were acquired using the FEI Tecnai T12 transmission electron microscope operated at 120 kV at a nominal magnification of 52,000x, corresponding to 2.14 Å/pixel at the specimen level. 2D classifications of picked particles were performed with CryoSPARC v4.6.2^12^. The detailed protocol for negative-stain examination of VPS13C and related constructs were deposited in protocol.io (doi.org/10.17504/protocols.io.rm7vz92d4gx1/v1).

#### Cryo-EM sample preparation and data collection

Cryo-EM grids were cleaned by an oxygen/argon plasma with Gatan Solarus Advanced Plasma System (Model 950). A 3.5 μL aliquot of concentrated VPS13C (~0.04 mg/ml) was applied to Quantifoil R1.2/1.3 300 mesh gold grids coated with a 2nm continuous carbon film (Cat. # 668–300-AU, Ted Pella), and incubated for 30 seconds. Grids were blotted for 1.5 seconds with a blot force of −12 using the Vitrobot Mark IV (Thermo Scientific) under 100% humidity at 8 °C, then plunge-frozen in liquid ethane cooled by liquid nitrogen. Pre-screened grids were stored in liquid nitrogen prior to loading into a Titan Krios transmission electron microscope (FEI) operated at 300 kV, equipped with a BioQuantum energy filter and a K3 direct electron detector (Gatan). Data acquisition was performed using SerialEM^42^ in super-resolution mode (0.534 Å/pixel) with a defocus range of −2.0 to −2.5 μm, which was selected during grid screening to facilitate particle picking. Each movie was recorded with an exposure time of 2.896 seconds and dose-fractionated into 50 frames, resulting in a total electron dose of 42.7 e^−^/Å^2^. A total of 4,140 movies were collected at 0° tilt and 4,914 movies at 25° tilt. The protocol of VPS13 structural determination has been deposited in protocol.io (doi.org/10.17504/protocols.io.36wgqpqpovk5/v1). Raw movies were deposited into EMPAIR (EMPIAR-13070).

#### Cryo-EM data processing

Cryo-EM data processing was carried out using CryoSPARC v4.6.2^12^ using Cryo-EM Cloud Infrastructure, recently developed for the SBGrid Consortium^47^. Beam-induced motion was corrected using patch motion correction with a Fourier crop factor of 0.5, resulting in a physical pixel size of 1.068 Å. Contrast transfer function (CTF) parameters were estimated using patch CTF estimation^12^. Following manual micrograph curation, 7,178 movies were selected for further processing. Approximately 2.6 million particles were picked using the blob picker with an elliptical blob (100 and 300 Å axis dimensions), and extracted with a box size of 430 pixels (unbinned). After three rounds of 2D classification, 579,426 particles were used to generate six ab-initio models. All particles were subjected to one round of heterogeneous refinement, and the best-resolved volume was further refined using non-uniform refinement. After one round of 3D classification and non-uniform refinement using 392,473 particles, the full-length map (map #1, EMD-73345) has a global resolution of 4.2 Å but the N-terminus remained largely unresolved. Particles were then re-picked using templates generated from the full-length volume with a 160 Å picking distance, yielding 2,040,737 particles. These particles were extracted with a box size of 512 pixels and down-sampled to 384 pixels. They were split into subsets and mixed with the original 579,426 particles for two rounds of heterogeneous refinement (classification). The highest-resolution class was selected to generate a C-terminal mask. After removing duplicate particles within 100 Å, 403,001 unique particles were subjected to local refinement with re-centering, resulting in a C-terminal map at 3.8 Å resolution (EMD-73344).

A separate round of template picking with a 96 Å separation yielded 4,203,081 particles. They were extracted with a 512-pixel box and down-sampled to 384 pixels. After two rounds of 2D classification, we selected a subset of 190,805 particle that displays improved alignment at the N-terminus. These particles were used for ab initio reconstruction and non-uniform refinement, producing a low-resolution volume representing the N-terminal domain, which was subsequently used to generate a local refinement mask. Simultaneously, the N-terminal volume served as an input volume for two rounds of heterogeneous refinement and one round of 3D classification using all 4,203,081 particles. From this, 461,163 particles were selected and subjected to local refinement with the gaussian prior turned off, yielding an N-terminal map at 4.1 Å resolution (EMD-73343).

All resolutions were estimated using the gold-standard Fourier shell correlation (FSC) 0.143 criterion with noise substitution^48,49^. Final reconstructions for the full-length, C-terminal, and N-terminal maps were post-processed using spIsoNet^45^ to correct for artifacts caused by preferred particle orientation.

#### Model building and refinement

Initial atomic models corresponding to the C-terminal and N-terminal regions of VPS13C in complex with calmodulin were generated individually using AlphaFold server^18^. Structural fragments from these AlphaFold models, including calmodulin, were manually fitted into the cryo-EM density maps in UCSF ChimeraX^41^. The models were rebuilt using the DeepMainMast algorithm^43^ and RosettaCM^50^. Rebuilt models were fitted into the EM densities using Namdinator^44^. Both N-terminal and C-terminal models were refined using real_space_refine in Phenix with geometry restraints, followed by manual adjustments in Coot and validation in Phenix^39,40^. The resulting models were deposited into PDB (PDB-9YQQ; PDB-9YQP).

To generate the full-length model, the N- and C-terminal maps were aligned and combined with the full-length map using the ‘vol max’ command in ChimeraX^41^ to generate a composite map (EMD-73373). The final N- and C-terminal models were docked into the composite map. The models for the missing two central RGB domains were generated using AlphaFold and fitted into the composite map. The complete full-length model was refined using Namdinator^44^. manually adjusted in Coot, and further refined and validated in Phenix^39,40^ (PDB-9YRP).

For the structure of CtVPS13α (EMDB-21113)^10^ in complex with calmodulin, an initial model was generated with AlphaFold3^18^ and fitted into the EM density in ChimeraX^41^. This model was refined using Namdinator^44^ and Phenix^40^, and manually adjusted in Coot^39^. Model validation was performed in Phenix^40^ (PDB-9YRM). All validation statistics were shown in Table S2. All structure figures were prepared using ChimeraX^41^. The process of CryoEM processing and model building have been deposited in protocol.io: doi.org/10.17504/protocols.io.36wgqpqpovk5/v1.

The buried surface area between CaM and VPS13C or *Ct*VPS13α was calculated in ChimeraX^41^. Specifically, the sum of surface areas buried in VPS13 and CaM was computed as SASA(CaM) + SASA(VPS13C) − SASA(VPS13-CaM complex), where SASA is the solvent accessible surface area.

#### Structural comparison of the lipid transfer domain among BLTPs

To compare the architecture of lipid transfer domains across BLTP family proteins, molecular models comprising the N-terminal cap and the first five RBG domains were aligned in ChimeraX with the lipid channel axis facing inward viewing from the N-terminal end. The specific domain boundaries used for alignment were as follows: VPS13A (2–3174), VPS13C (2–3753), *Ct*VPS13 α (2–1231), ATG2A (173–1414; PDB: 8KBY), and BLTP-1 (65–1308; PDB: 9CAP). To enable a focused view of the lipid bridge domain, adaptor domains and flexible loops were removed from all models. Each structure was clipped from the N-terminus using a thin plane perpendicular to the channel axis, guided by the *Side View* module in ChimeraX^41^. The camera view and clipping plane were fixed relative to each other to maintain consistent imaging depth and magnification across all sections in every model. Starting from the N-terminus, the clipping plane was moved in 5 Å increments along the channel axis. Each slice was exported as a separate image, normalized to identical dimensions and pixel size. To estimate the width and the cross-sectional area of the channel, slice images were analyzed in ImageJ^38^. Real-space dimensions were calibrated in ChimeraX^41^ using the *Measure and Color Blobs* module on three representative clips. These measurements were then applied to quantify channel geometry across all slices. Data were plotted in GraphPad Prism 6 (RRID:SCR_002798). The alignment of BLTPs’ models and their comparisons were deposited in Zenodo (doi.org/10.5281/zenodo.17417584).

#### Mass spectrometry analysis for CaM identification

Full-length human VPS13C (UniProt Q709C8) fused to a C-terminal GFP-3×FLAG tag was expressed in Expi293F cells (Gibco) and purified as described. The protein was eluted with Buffer A supplemented with 0.25 mg/mL FLAG peptide, and the eluate was analyzed by mass spectrometry at the W. M. Keck Foundation Biotechnology Resource Laboratory, Yale School of Medicine. The list of proteins co-purified with VPS13C is shown in Table S2.

#### Western blotting

To examine the calmodulin co-purified with full-length VPS13C, VPS13C mutants, and *Ct*VPS13 in various buffer conditions, eluted proteins were resolved on 4–20% Tris-glycine precast gels. Proteins were transferred to PVDF membranes at 90 V for 90 minutes at 4°C in transfer buffer (25 mM Tris, 192 mM glycine, and 20% methanol). Membranes were blocked for 1 hour at room temperature in 3% BSA prepared in Tris-buffered saline containing 0.1% Tween-20 (TBST), then incubated overnight at 4 °C with the anti-calmodulin antibody (Cat. #HY-P82082, MCE), 1:1000 diluted in 3% BSA and TBST. The next day, membranes were washed three times with TBST, incubated for 1 hour at room temperature with the goat anti-rabbit HRP conjugate (Cat. #AP307P, Sigma Aldrich) at 1:1000 dilution. Membranes were washed another three times with TBST and incubated with ECL substrates (Cat. #32106, Thermo Scientific) prior to imaging by ImageQuant LAS 4000. The protocol has been deposited in protocols.io (doi.org/10.17504/protocols.io.kqdg31w17l25/v1).

To confirm the full-length VPS13C and the VAB domain of VPS13C were co-purified with Rab7A(QL), proteins were resolved on the 3–8% Tris-acetate precast gels and subjected to western blot following the above-mentioned procedures. The anti-FLAG primary antibody (Cat. #F1804, Sigma Aldrich) and anti-mouse HRP conjugate (Cat. #62–6520, Thermo Scientific) were both used at 1:1000 dilutions.

To confirm all calmodulin-binding defective mutants have similar expression levels, same quantity of Expi293F cell lysates expressing each mutant was loaded on 3–8% Tris-acetate precast gels and later blot for FLAG (Cat. #F1804, Sigma Aldrich) and α-Tubulin (Cat. #2125, CST) with corresponding secondary HRP conjugates.

To examine the quality of the ATG2_C domain of VPS13C, lysates from Expi293F cells expressing FLAG–mCherry–ATG2_CVPS13C were loaded on 4–20% Tris–glycine precast gels (Bio-Rad) and transferred to nitrocellulose membranes (Cat. #1620146, Bio-Rad). The membranes were probed with anti-FLAG antibody (Cat. #F1804, Sigma Aldrich), followed by secondary antibodies conjugated to IRDye 800CW (LI-COR; 1:10,000), and imaged using the Odyssey imaging system (LI-COR) according to the manufacturer’s instructions.

To examine the amount of VPS13C co-immunopurified with FLAG-VAPA, lysates from HEK293T cells were loaded on 1mm thick 4–20% Tris–glycine WedgeWell precast gels (Cat. #XP04205, Thermo Scientific) and transferred to nitrocellulose membranes (Cat. #1620146, Bio-Rad). Membranes were blocked for 1 hour at room temperature in 5% milk (Cat. # M17200, Research Products International) prepared in Tris-buffered saline containing 0.1% Tween-20 (TBST), then incubated overnight at 4 °C with the anti-VPS13C antibody (Cat. # 29844–1-AP, Proteintech; 1:1,000), anti-Alpha Tubulin (Cat. #T5168, Sigma Aldrich; 1:5,000) or anti-FLAG (Cat. #F3165, Sigma Aldrich; 1:5,000), followed by secondary antibodies conjugated to IRDye 800CW (LI-COR; 1:10,000), and imaged using the Odyssey imaging system (LI-COR) according to the manufacturer’s instructions.

#### Expression and purification of *Ct*VPS13(1–635)

Both pETDuet-6xHis-Strep-*Ct*VPS13(1–635) and pETDuet-6xHis-Strep-*Ct*VPS13(1–635)-6xHis-*Hs*CaM constructs were transformed to BL21(De3) pLys cells. For both constructs, 1 liter of Luria Broth (LB) culture was grown to an OD600 of 0.8, induced with 0.5mM IPTG (Cat. #I56000, RPI) and harvested after culturing at 18°C for 16 hours. Cells were resuspended in buffer A, supplemented with 5mM imidazole (Cat. #56750, Sigma Aldrich). The suspension was passed 5 times through Avestin EmulsiFlex-C5 operating at 7,500 psi followed by a centrifugation step at 36,945 rcf for 30 minutes at 4°C. The supernatant was incubated in batch with Talon metal affinity Resin (Cat. #635502, Takara) for 30 minutes while rotating at 4°C. Affinity resin was washed with 50 column volumes of buffer A containing 20mM imidazole and protein was eluted using buffer A containing 300 mM imidazole. The protocol has been deposited in protocols.io (doi.org/10.17504/protocols.io.ewov11m17vr2/v1).

#### Cell culture, transfection, live-cell imaging, and immunoprecipitation (IP)

For live-cell imaging experiments, cells were seeded onto glass-bottomed dishes (MatTek). Following incubation for 24 h, the cells were transfected using FuGene HD (Cat. #E2311, Promega) according to the manufacturer’s recommendations. Spinning-disk confocal imaging was performed 16–24 h post transfection. Growth media were changed with live-cell imaging solution (Cat. #A59688DJ, Invitrogen) shortly before imaging. Imaging was performed at 37 °C in 5% CO2 using a Nikon Ti2-E inverted microscope equipped with a Spinning Disk Super Resolution by Optical Pixel Reassignment Microscope (Yokogawa CSU-W1 SoRa, Nikon) and Microlens-enhanced Nipkow Disk with pinholes and a ×60 SR Plan Apo IR oil-immersion objective. For LLOMe (Cat. #L7393, Sigma Aldrich) and thapsigargin (TG) (Cat. #T9033, Sigma Aldrich) experiments, a 2x stock solution was prepared in the imaging solution and added to the dish during image acquisition. A final concentration of either 1 mM LLOMe or 1 μM thapsigargin was used. A detailed description of cell culture, transfection and imaging is available at doi.org/10.17504/protocols.io.eq2lyp55mlx9/v1.

To generate RPE-1 cells stably expressing VPS13ĈmStayGold, the PiggyBac transposon system was used. Full-length human VPS13ĈmStayGold was cloned into a PiggyBac transposon vector under the CAG promoter, with a Zeocin (Cat. #R25005, Gibco) resistance cassette for selection. This plasmid was co-transfected with a PiggyBac transposase expression vector into RPE-1 cells, and a pool of stable integrants were selected and maintained in media supplemented with Zeocin. A detailed description of piggybac cell line generation is available at doi.org/10.17504/protocols.io.yxmvm9nr5l3p/v1.

For the FLAG-VAPA IP experiments, HEK293T cells were seeded in 10-cm plates and cultured for 24 hours, followed by transfection with FuGene 4K Transfection Reagent (Cat. #E5911, Promega). After 24 hours, the growth medium was replaced with 1 μM thapsigargin or DMSO in live-cell imaging buffer. After 1 minute of treatment, cells were pelleted and flash-frozen in liquid nitrogen. Anti-FLAG immunoprecipitation was performed using Anti-FLAG M2 magnetic beads (Cat. #M8823, Millipore) according to the manufacturer’s instructions. Bound proteins were eluted and analyzed by western blotting.

#### HDX-MS sample preparation

HDX reactions comparing VPS13C-dATG2C-3xFLAG/CaM and EGTA to VPS13C-dATG2C-3xFLAG/CaM and Ca^2+^ were carried out in a 12.0μL reaction volume containing 12 pmol of VPS13C-dATG2C-3xFLAG/CaM and 2mM of either EGTA or Ca^2+^. The exchange reactions were initiated by the addition of 8.0 μL of D_2_O buffer (50 mM HEPES pH 7.2, 200mM NaCl, 1mM TCEP, 90.7%D_2_O(V/V)) to 4.0 μL of protein (final D2O concentration of 56.7%). Reactions proceeded for 3s, 30s, and 300s at 18°C before being quenched with ice cold acidic quench buffer, resulting in a final concentration of 0.6M guanidine HCl and 0.9% formic acid post quench. All conditions and timepoints were created and run in independent triplicate. Samples were flash frozen immediately after quenching and stored at −80°C until injected onto the ultra-performance liquid chromatography (UPLC) system for proteolytic cleavage, peptide separation, and injection onto a QTOF for mass analysis, described below.

#### Protein Digestion and MS/MS Data Collection

Protein samples were rapidly thawed and injected onto an integrated fluidics system containing a HDx-3 PAL liquid handling robot and climate-controlled (2°C) chromatography system (Trajan), a Acquity UPLC I-Class Series System (Waters), as well as an Impact II QTOF Mass spectrometer (Bruker). The full details of the automated LC system were previously described in^51^. The samples were run over an immobilized pepsin column (Affipro; Cat. #AP-PC-001) at 200 μL/min for 4 minutes at 2°C. The resulting peptides were collected and desalted on a C18 trap column (Acquity UPLC BEH C18 1.7 μm column (2.1 × 5 mm); Waters 186004629). The trap was subsequently eluted in line with an ACQUITY 300Å, 1.7 μm particle, 100 × 2.1 mm BEH C18 UPLC column (Waters), using a gradient of 3–10% B (Buffer A 0.1% formic acid; Buffer B 100% acetonitrile) over 1.5 minutes, followed by a gradient of 10–25% B over 4.5 minutes, followed by a gradient of 25–35% B over 5 minutes, finally after 1 minute at 35% B a gradient of 35–80% B over 1 minute was used. Mass spectrometry experiments acquired over a mass range from 150 to 2200 m/z using an electrospray ionization source operated at a temperature of 200°C and a spray voltage of 4.5 kV.

#### Peptide identification

Peptides were identified from the non-deuterated samples of VPS13C-dATG2C-3xFLAG/CaM and VPS13C-3xFLAG using data-dependent acquisition following tandem MS/MS experiments (0.5 s precursor scan from 150–2000 m/z; twelve 0.25 s fragment scans from 150–2000 m/z). MS/MS datasets were analyzed using FragPipe v23.1 and peptide identification was carried out by using a false discovery-based approach using a database of purified proteins and known contaminants^52–54^. MSFragger was utilized, and the precursor mass tolerance error was set to −20 to 20ppm. The fragment mass tolerance was set at 20ppm. Protein digestion was set as nonspecific, searching between lengths of 4 and 50 aa, with a mass range of 400 to 5000 Da.

#### Mass Analysis of Peptide Centroids and Measurement of Deuterium Incorporation

HD-Examiner Software (Trajan) was used to automatically calculate the level of deuterium incorporation into each peptide. All peptides were manually inspected for correct charge state, correct retention time, appropriate selection of isotopic distribution, etc. Deuteration levels were calculated using the centroid of the experimental isotope clusters. Results are presented as relative levels of deuterium incorporation and the only control for back exchange was the level of deuterium present in the buffer (56.7%). Differences in exchange in a peptide were considered significant if they met all three of the following criteria: ≥5% change in exchange, ≥0.40 Da difference in exchange, and a p value <0.01 using a two tailed student t-test. Samples were only compared within a single experiment and were never compared to experiments completed at a different time with a different final D2O level. The data analysis statistics for all HDX-MS experiments are in the HDX Stats table in the source data file according to published guidelines.^55^ The mass spectrometry proteomics data have been deposited to the ProteomeXchange Consortium via the PRIDE partner repository^56^ with the dataset identifier (PXD076522). The detailed protocol of HDX-MS analysis of the VPS13C-CaM complex has been deposited in protocols.io (doi.org/10.17504/protocols.io.eq2ly4q4qlx9/v1).

#### Strep pull-down of VPS13C mutants by Strep-Rab7A-Q67L

The proteins were co-expressed and purified from Expi293 cells as mentioned above. For co-transfection, 30 μg of plasmid encoding N-terminally Strep-tagged Rab7A and 70 μg plasmid encoding C-terminally FLAG-tagged VPS13C FL or mutants were mixed in Opti-MEM (Cat. #31985070, Gibco), and transfected into 100 mL of Expi293F cells. Following 48 hours of expression, cells were harvested and resuspended in 10 mL buffer A supplemented with 1 mM GTP (Cat. # G8877, Sigma Aldrich) and 2 mM MgCl_2_, then lysed by three cycles of freeze-thaw. After lysate clarification, the supernatant was divided, with 30% subjected to FLAG pull down using 50 μL anti-Flag M2 resin. The remaining 70% was used for Strep pull down with 50 μL Strep-Tactin XT 4Flow high-capacity resin (Cat. #2-5030-002, IBA) for 2 hours at 4°C. The resin was washed four times with 15 mL buffer A. Elution was performed three times, each in 50 μL buffer A supplemented with 0.5 mg/mL FLAG peptide for FLAG pull down or 50 mM D-Biotin (Cat. #BG-00, G-Biosciences) for Strep pull down and incubating for 20 minutes. Eluates were pooled and concentrated to a final volume of 50 μL. For protein quantification, Eluates from the Strep pull down were analyzed by SDS-PAGE. Rab7A was quantified on 4–20% Tris-glycine precast gels (Cat. #4561096, Bio-Rad). And the co-purified VPS13Cs were resolved on 3–8% Tris-acetate precast gels (Cat. #EA0375BOX, Invitrogen). The amount of co-purified VPS13C was normalized to the quantity of Rab7A to evaluate relative binding affinity among VPS13C mutants. The VAB domain alone of VPS13C that was co-purified with Rab7A cannot be quantified by Coomassie Blue, yet detectable by anti-FLAG western blot. For quantification and comparison, the band intensity of each VPS13C construct retained by Rab7 was normalized by the corresponding Rab7 band intensity, and then normalized to the full-length (FL) construct. Four biological replicates were performed as independent experiments, except ΔC+WWE, where two replicates were performed. Data were plotted as mean ± s.d. and compared using a two-sided Student’s t-test. The protocol of VPS13C-Rab7A Co-IP has been deposited in protocols.io(doi.org/10.17504/protocols.io.eq2ly4q4qlx9/v1).

#### Cross-linking of VPS13C and Rab7A(Q67L)

The MBP-TEV-Rab7A-6xHis construct was expressed in Expi293F cells and purified following the same protocol used for VPS13C with a few modifications. For MBP-affinity purification, the clarified lysate was incubated with Amylose resin (Cat. #E8021, NEB) for 2 hours at 4°C, washed, and proteins were eluted with buffer A supplemented with 50 mM maltose (Cat. #M5895, Sigma Aldrich). Pooled eluates were run into the Superose 6 size exclusion column in buffer B to remove maltose. For cross-linking, purified full-length VPS13C-3xFLAG and MBP-Rab7A-6xHis were mixed at a 1:10 molar ratio, and incubated overnight at 4°C. Cross-linking was initiated by adding 0.1% glutaldehyde to VPS13C alone, Rab7A alone, or the VPS13C+Rab7A mixture, followed by incubation for 15 minutes at room temperature. The reactions were quenched with 100 mM Tris (pH 8.0) for 10 minutes at room temperature. All samples were subject to MBP pull down as described above. The VPS13C cross-linked to Rab7A was analyzed by SDS-PAGE. The detailed protocol of VPS13C-Rab7A complex formation has been deposited in protocols.io (doi.org/10.17504/protocols.io.eq2ly4q4qlx9/v1).

#### Preparation of SUVs

POPC (Cat. #850457), POPE (Cat. #850757), DGS-NTA (Ni) (Cat. #790404), and Lissamine Rhodamine B-PE (Rhod-PE; Cat. #810150) were purchased from Avanti Polar Lipids. Lipids were mixed at a molar ratio of 84.8% POPC, 10% POPE, 5% DGS-NTA and 0.2% Rhod-PE. The mixture was dried under a gentle nitrogen stream and desiccated under vacuum overnight. The dried lipid film was rehydrated in buffer D (200 mM NaCl, 25 mM HEPES, pH 7.5, 1 mM TCEP) to a final concentration of 10 mM. The suspension was incubated at 37°C for 60 minutes with gentle mixing every 20 minutes, followed by five freeze-thaw cycles. The liposomes were then extruded 31 times through a 200 nm polycarbonate filter using the Avanti Mini-Extruder to generate SUVs. The protocol of liposome preparation has been deposited in protocols.io (doi.org/10.17504/protocols.io.q26g7n9nqlwz/v1).

#### Flotation assay of SUVs

In a 100 μL reaction, 1–5 μg purified full-length VPS13C or VPS13C-ΔATG2C was mixed with 1 mM SUVs. For experiments where Rab7A(QL)-6xHis was used, the protein was produced by TEV cleavage of the MBP-TEV-Rab7A-6xHis construct and further purification through size-exclusion chromatography. Alternatively, the Rab7A(Q67L/T22N)-6xHis was produced from bacteria as above for the *Ct*VPS13(1–635) construct. Equal mass of Rab7A(Q67L/T22N)-6xHis or 6xHis-ATG7 (negative control, gift from the Melia Lab, Yale University) was added with VPS13C-ΔATG2C to SUVs in the presence of 1 mM GTP and 2 mM Mg^2+^. The mixture was incubated at room temperature for 20 minutes.

For the floatation assay, a 90 μL portion of the mixture was combined with equal volume of 60% OptiPrep (Cat. #D1556, Sigma Aldrich) to generate the 30% layer, and transferred to a 0.8 mL ultracentrifuge tube (Cat. #344090, Beckman). The sample was layered with 300 μL of 20% OptiPrep, followed by 100 μL of reconstitution buffer. The gradient was centrifuged at 193,911xg for 60 minutes in a SW-55 Ti rotor. After centrifugation, 100 μL from the top fraction, which contains all floated SUVs was recovered. A 10 μL input, along with the 30% of the top fraction was analyzed by SDS-PAGE on 3–8% Tris-acetate precast gels and quantified using ImageJ. For quantification, the normalized quantity of proteins in the top fraction was divided by the normalized quantity of proteins from the input, and shown as a percentage. Four biological replicates were performed as independent experiments for FL and ΔATG2C constructs; three biological replicates were performed for other samples. Data were shown as mean ± s.d. and compared using a two-sided Student’s t-test. The detailed protocol of SUV-related experiments has been deposited in protocols.io (doi.org/10.17504/protocols.io.q26g7n9nqlwz/v1).

#### Preparation of giant unilamellar vesicles (GUVs)

GUVs were generated as previously described^57^ with a few modifications. Specifically, coverslips were sonicated in distilled water and ethanol, then incubated at room temperature in 10% APTES ((3-aminopropyl) trimethoxysilane; Cat. #A3648, Sigma Aldrich) in ethanol for 30 min. After incubation, coverslips were washed with ethanol and distilled water, followed by incubation in 2.5% glutaraldehyde (GA; Cat. #G6257, Sigma Aldrich) in PBS for 30 min. Coverslips were washed in distilled water, dried, and stored at 4 °C. Then, PAA gels were prepared from acrylamide (AA; 40% w/v, Sigma Aldrich, A4058) and N,N′-methylenebisacrylamide (BAA; 2% w/v, Cat. #M1533, Sigma Aldrich) stock solutions. A 1 mL gel solution contained 250 μL acrylamide, 10 μL bis-acrylamide, and 729 μL H_2_O. Solutions were degassed in a vacuum chamber for 30 minutes before polymerization. Polymerization was initiated by adding 10 μL ammonium persulfate (APS; Cat. #248614, Sigma Aldrich) and 1 μL N,N,N′,N′-tetramethylethylenediamine (TEMED; Cat. #1610801, Bio-Rad). 30 μL of the solution was placed on a clean glass plate, and the prepared coverslips were placed on top, ensuring full contact. After 15 minutes of polymerization, coverslips were carefully removed with a razor blade, washed with water, and dried at 50 °C for 30 min. To generate GUVs, all lipids were purchased from Avanti Polar Lipids. Lipid mixtures were dissolved in chloroform at 1 mg/mL in glass vials. The lipid compositions (molar %) were: POPC-GUVs: 89.5% POPC, 10% DOPS (Cat. #840035, Avanti Polar Lipids), 0.5% Rhod-PE or Cy5-DOPE (Cat. #810335, Avanti Polar Lipids). DOG-GUVs: 59.5% POPC, 10% DOPS, 30% DOG (Cat. #800811, Avanti Polar Lipids), 0.5% Rhod-PE or Cy5-DOPE. Coverslips were placed in 6-well plates, and 20 μL of lipid solution was applied onto each gel. A glass Drigalski spatula was used to evenly spread the solution. The solvent was evaporated in a vacuum chamber for 30–40 min. 1 mL of buffer (500 mM sucrose, 25 mM HEPES, pH 7.4) was carefully added to the wells and incubated overnight. Vesicles were collected by gently pipetting the solution, repeatedly aspirating and dispensing, and transferred to clean 1.5 mL tubes.

#### Confocal fluorescence microscopy of protein–GUV association

GUVs (1 μL) were incubated with either 4 μL purified proteins (300 nM working concentration) or cell lysates in 35 mm glass-bottom MatTek dishes (pre-passivated with BSA) at room temperature for 10 min. Images were acquired using the same Spinning Disk Super Resolution by Optical Pixel Reassignment Microscope described above. All images were analyzed with ImageJ. The detailed protocol of GUV-related experiments has been deposited in protocols.io (doi.org/10.17504/protocols.io.81wgbwko1gpk/v1).

#### C-laurdan staining and Generalized Polarization (GP) analysis

C-laurdan (Cat. #HY-D1378, MCE) GP measurements in GUV were performed similarly to described.^58^ C-laurdan (20mM in DMSO) was prediluted in the protein buffer (500mM HEPES, pH 7.2, 200mM NaCl, 1mM DTT) to 20 μM and mixed with POPC-GUVs or DOG-GUVs at a 4:1 ratio at room temperature for 30 minutes. GUVs were imaged in 35 mM glass-bottom MatTek dishes (pre-passivated with BSA). Images were acquired on a Zeiss LSM 880 confocal microscope equipped with a Quasar Spectral detector with 32-channel GaAsP PMT. C-laurdan was excited with a 405 nm laser, and fluorescence in the 420–460 nm (I440) and 470–510 nm (I490) ranges were collected simultaneously by the spectral detector. To calibrate the relative sensitivity of the two channels, the standard GP_exp_ value for 20 mM C-laurdan in DMSO was calculated according to equation 1^59^, using the raw integrated densities were calculated in ImageJ^38^. The G factor was calculated to be 1.352 according to equation 2, using GP_exp_ = 0.207, as widely used for Laurdan.^60,61^

(Equation 1) $$GP_{\exp}=\frac{I_{Ch1}-I_{Ch2}}{I_{Ch1}+I_{Ch2}}$$

(Equation 2) $$G=\frac{GP_{\mathrm{theo}}+GP_{\mathrm{theo}}\times GP_{\exp}-GP_{\exp}-1}{GP_{\exp}+GP_{\mathrm{theo}}\times GP_{\exp}-GP_{\mathrm{theo}}-1}$$

Using the same microscopy setup, a total of 26 micrographs for the POPC sample and 21 micrographs for the POPC+DOG sample were recorded in 8-bit format with 512 × 512 pixels. To prevent GUV rupture, all images were acquired with a short exposure time. Analysis of GP values per micrograph was carried out manually in ImageJ by recording the total density around the region of interest (GUVs), followed by computing GP values according to equation 3. For comparison, Welch’s t-test was performed in GraphPad Prism.

Alternatively, GP values per pixel was analyzed with a customized Python script (doi.org/10.5281/zenodo.19688024). To increase signal-to-noise ratio, all images were binned four times to 128 × 128 pixels, and pixels below 20% of the maximum signal of the image were discarded. GP values per pixel were computed according to equation 3. The per pixel GP values for all POPC or POPC+DOG micrographs were pooled and subjected to Welch’s t-test for comparison in GraphPad Prism.


(Equation 3) $$GP_{\mathrm{corr}}=\frac{I_{Ch1}-G\times I_{Ch2}}{I_{Ch1}+G\times I_{Ch2}}$$

#### Quantification and Statistical Analysis.

Statistical analysis was performed using GraphPad Prism v6.01 or v11.0. Statistical details for each experiment, including the exact value of n, what n represents, definition of center, error bars, statistical tests, and significance levels, are provided in the corresponding figure legends. Unless otherwise noted, data are presented as mean ± s.d. For C-Laurdan generalized polarization measurements (Fig. S6), data are presented as mean ± 95% confidence interval to show the precision of the mean. Statistical significance was defined as P < 0.05; n.s., not significant; *P < 0.05; **P < 0.01; ***P < 0.001; ****P < 0.0001. Comparisons between two groups were performed using two-sided t-test, as indicated in the figure legends. No statistical method was used to predetermine sample size. Randomization was not used, as experiments involved biochemical reconstitution and image-based quantification of pre-defined samples. No data were excluded. Assumptions of the statistical tests were not formally tested.

## Supplementary Material

1

2 Table S2: Mass-spec analysis of proteins that co-purified with VPS13C-3xFLAG, related to Figure 2.

3 Video SV1: Thapsigargin-induced calcium elevation does not alter VPS13C localization, related to Figure 2 and S5. RPE-1 cells expressing VPS13ĈmStayGold (green) and the calcium indicator R-GECO (magenta) were imaged every 5 seconds. 1 μM thapsigargin (TG) was added at t = 35 s (frame 8). The movie plays at 7 frames per second (fps). Scale bars, 10 μm. Still images from this movie are shown in Fig. S5A.

4 Video SV2: LLOMe-induced lysosomal recruitment of VPS13C, related to Figure 2 and S5. RPE-1 cells stably expressing VPS13ĈmStayGold were imaged every 1 minute. 1 mM LLOMe was added after the first frame. The movie plays at 7 frames per second (fps). Scale bars, 10 μm. Still images from this movie are shown in Fig. S5B.

5 Video SV3: Thapsigargin-induced calcium elevation in HEK293T cells, related to Figure 2 and S5. HEK293T cells expressing the calcium indicator R-GECO (magenta) were recorded. Thapsigargin (TG) was added to a final concentration of 1 μM at t = 6 s. The movie plays at 30× speed. Scale bars, 20 μm. The quantification of the fluorescence fluctuation is shown in Fig. S5C.

6 Video SV4: Thapsigargin-induced calcium elevation does not alter the localization of an N-terminal fragment of VPS13C, related to Figure 2 and S5. RPE-1 cells expressing N-terminal fragment VPS13C_(1–1390)_-mStayGold (green) and the calcium indicator R-GECO (magenta) were imaged every 5 seconds. 1 μM thapsigargin (TG) was added at t = 15 s (frame 4). The movie plays at 7 frames per second (fps). Scale bars, 10 μm. Still images from this movie are shown in Fig. S5F.

## Figures and Tables

**Table 1.** Cryo-EM Data Collection and Model Statistics.

Description	*Hs*VPS13C with co-purified human calmodulin				*Ct*VPS13α
	C-terminal	N-terminal	Full-length	Composite	
	PDB:9YQQ	PDB:9YQP		PDB:9YRP	PDB:9YRM
	EMDB:73344	EMDB:73343	EMDB:73345	EMDB:73373	EMDB:21113
**Data Collection and Processing**					
Facility	Yale West Campus-Cryo-EM facility				EMDB-21113
Microscope	Titan Krios				
Voltage (kV)	300				
Camera	K3				
Magnification	81,000				
Pixel Size (Å)	0.534 (super resolution)				
Total Electron Exposure (e-/A^2^)	43				
Defocus Range (μm)	2–2.5				
Symmetry Imposed	C1				
Num of mics	7178				
Initial Particles	2,040,737	4,203,081	2,072,307		
Final Particles	403,001	461,163	392,473		
				
**Refinement**					
Initial models	AlphaFold	AlphaFold	AlphaFold and N and C-terminal models		
Map pixel size	1.424	1.424	1.424	1.424	
Map Resolution (Å) (FSC 0.143)	3.80	4.10	4.13		
Map sharpening B-factor (Å^2^)	−111.65	−106.52	−147.66		
					
**Model Composition**					
Non-hydrogen atoms	13839	8956		24677	9116
Protein residues	1739	1118		3092	1128
Ligands	0	0		0	0
					
**Model vs. Data**					
FSC Map to Model (Å) (FSC 0.5)	6.9	7.5		7.4	4.2
Correlation coefficient (mask)	0.58	0.55		0.61	0.71
					
**B factors (A^2^)**					
Protein	20.01	8.20		74.47	63.62
					
**R.m.s deviation**					
Bond length (Å)	0.004	0.004		0.004	0.004
Bond angles (°)	1.006	1.006		1.002	1.007
					
**Validation**					
Molprobity score	1.85	1.99		1.90	1.74
Clashscore	6.64	8.34		8.01	6.03
Rotamer outliers (%)	0.06	0.00		0.00	0.40
		
**Ramachandran plot**					
Outliers (%)	0.12	0.00		0.00	0.00
Allowed (%)	7.86	9.73		7.44	6.14
Favored (%)	92.02	90.27		92.56	93.86
**Rama-Z (whole)**	−2.25	−1.06		−1.69	−0.82

**Key Resource Table:** 

REAGENT or RESOURCE	SOURCE	IDENTIFIER
Antibodies		
Rabbit anti-calmodulin	Med Chem Express	Cat. #HY-P82082; RRID:AB_3104023
Goat anti-rabbit HRP conjugate	Sigma Aldrich	Cat. #AP307P; RRID:AB_92641
Mouse anti-FLAG	Sigma Aldrich	Cat. #F1804; RRID:AB_262044
Goat anti-mouse HRP conjugate	Thermo Scientific	Cat. #62-6520; RRID:AB_88369
Rabbit anti α-Tubulin	Cell Signaling Technology	Cat. #2125; RRID:AB_2619646
Mouse anti-FLAG	Sigma Aldrich	Cat. #F3165;RRID:AB_259529
Mouse anti-Alpha Tubulin	Sigma Aldrich	Cat. #T5168; RRID:AB_477579
Rabbit anti-VPS13C	Proteintech	Cat. #29844-1-AP; RRID:AB_3086177
Goat anti-mouse antibody conjugated to IRDye 800CW	LI-COR	Cat. #926-32350; RRID:AB_2782997
Bacterial and virus strains		
BL21(DE3) pLysS	Agilent	Cat. #200132
Biological samples		
Chemicals, peptides, and recombinant proteins		
3xFLAG peptide	Apex Bio	Cat. #A6002
His-ATG7	Gift from Thomas Melia Lab, Yale University	N/A
Recombinant calmodulin	G-Biosciences	Cat. #786-1244
GTP (guanosine triphosphate)	Sigma Aldrich	Cat. #G8877; CAS: 36051-31-7
IPTG; Isopropyl-β-D-thiogalactopyranoside	Research Products International	Cat. #I56000; CAS: 367-93-1
Thapsigargin	Sigma Aldrich	Cat. #T9033; CAS: 67526-95-8
LLOME; Leu-Leu methyl ester hydrobromide	Sigma Aldrich	Cat. #L7393; CAS: 16689-14-8
Imidazole	Sigma Aldrich	Cat. #56750; CAS: 288-32-4
D-Biotin	G-Biosciences	Cat. #BG-00; CAS: 58-85-5
Maltose	Sigma Aldrich	Cat. #M5895; CAS: 6363-53-7
Glutaraldehyde (GA)	Sigma Aldrich	Cat. #G6257; CAS: 111-30-8
OptiPrep (iodixanol solution)	Sigma Aldrich	Cat. #D1556; CAS: 92339-11-2
Rhodamine B-PE (Rhod-PE)	Avanti Polar Lipids	Cat. #810150; CAS: 384833-00-5
POPC (1-palmitoyl-2-oleoyl-sn-glycero-3-phosphocholine)	Avanti Polar Lipids	Cat. #850457; CAS: 26853-31-6
POPE (1-palmitoyl-2-oleoyl-sn-glycero-3-phosphoethanolamine)	Avanti Polar Lipids	Cat. #850757; CAS: 26662-94-2
DGS-NTA(Ni)	Avanti Polar Lipids	Cat. #790404; CAS: 231615-77-3
DOPS (1,2-dioleoyl-sn-glycero-3-phospho-L-serine)	Avanti Polar Lipids	Cat. #840035; CAS: 90693-88-2
Cy5-DOPE (Cyanine-5 DOPE)	Avanti Polar Lipids	Cat. #810335; CAS: 2260669-61-0
DOG (1,2-dioleoyl-sn-glycerol)	Avanti Polar Lipids	Cat. #800811; CAS: 24529-88-2
Ammonium persulfate (APS)	Sigma Aldrich	Cat. #248614; CAS: 7727-54-0
TEMED (N,N,N’,N’-Tetramethylethylenediamine)	Bio-Rad	Cat. #1610801; CAS: 110-18-9
Acrylamide (AA; 40% w/v)	Sigma Aldrich	Cat. #A4058; CAS: 79-06-1
N,N’-Methylenebisacrylamide (BAA; 2% w/v)	Sigma Aldrich	Cat. #M1533; CAS: 110-26-9
(3-Aminopropyl)trimethoxysilane (APTES)	Sigma Aldrich	Cat. #A3648; CAS: 919-30-2
DMEM (Dulbecco’s Modified Eagle Medium)	Gibco	Cat. #11965092
Fetal bovine serum (FBS)	Gibco	Cat. #A5256701
Zeocin	Gibco	Cat. #R25005
GlutaMAX	Gibco	Cat. #3505006
Dry powder milk	Research Products International	Cat. # M17200
Anti-FLAG M2 magnetic beads	Millipore-Sigma	Cat. #M8823
Protease inhibitor cocktail	Roche	Cat. #11873580001
Opti-MEM reduced serum medium	Gibco	Cat. #31985070
Expi293 expression medium	Gibco	Cat. #A1435101
Talon metal affinity resin	Takara	Cat. #635502
Amylose resin	NEB	Cat. #E8021
Strep-Tactin XT 4Flow high-capacity resin	IBA	Cat. #2-5030-002
Anti-FLAG M2 resin	Millipore-Sigma	Cat. #A2220
Luria Broth	Research Products International	Cat. #L24340
Critical commercial assays		
FuGene HD Transfection Reagent	Promega	Cat. #E2311
FuGene 4K transfection reagent	Promega	Cat. #E5911
Live cell imaging solution	Invitrogen	Cat. #A59688DJ
ExpiFectamine 293 reagent	Gibco	Cat. #A14525
ECL substrate	Thermo Scientific	Cat. #32106
4-20% Tris-glycine WedgeWell precast gels	Thermo Scientific	Cat. #XP04205
Nitrocellulose membranes	Bio-Rad	Cat. #1620146
Deposited data		
Raw Cryo-EM data	This study	EMPIAR-13070
All raw datasets (Microscopy images, western blot, SDS-PAGE) generated in this study	This study	Mendeley Data - https://doi.org/10.17632/xgts777pxc.1
All raw datasets (Microscopy images, western blot, SDS-PAGE) generated in this study	This study	https://doi.org/10.5281/zenodo.17417584
Alternate key resource table	This study	https://doi.org/10.5281/zenodo.17459272
Cryo-EM map of the N-terminal VPS13C	This study	EMD-73343
Model built for N-terminal VPS13C	This study	PDB-9YQP
Cryo-EM map of the C-terminal VPS13C	This study	EMD-73344
Model built for C-terminal VPS13C	This study	PDB-9YQQ
Consensus Cryo-EM map of full-length VPS13C	This study	EMD-73345
Composite map of full-length VPS13C	This study	EMD-73373
Model built for composite full-length VPS13C	This study	PDB-9YRP
Model built for EMDB-21113, VPS13(1-1390) from C.thermphilium	This study	PDB-9YRM
Cryo-EM map for VPS13(1-1390) from C. thermophilum	Li et al.^10^	EMDB-21113
Experimental models: Cell lines		
HeLaM cells	Cellosaurus	Cat. #RCB5388; RRID: CVCL_R965
Expi293F cells	Cellosaurus	Cat. #A14527; RRID:CVCL_D615
hTERT-RPE1 cells	Cellosaurus	Cat. #CRL-4000; RRID:CVCL_4388
hTERT-RPE1 cells stably expressing VPS13C-WT-FL-mStayGold	Cellosaurus	RRID:CVCL_F4CL
HEK293T cells	Cellosaurus	Cat. #CRL-3216, RRID:CVCL_0063
Experimental models: Organisms/strains		
Recombinant DNA		
pCAG-VPS13C-WT-3xFLAG	This study	RRID:Addgene_248315
pCAG-VPS13C-delATG2C-3xFLAG	This study	RRID:Addgene_248316
pCAG-VPS13C-delC-3xFLAG	This study	RRID:Addgene_248317
pCAG-VPS13C-delVAB-3xFLAG	This study	RRID:Addgene_248318
pCAG-VPS13C-delCdelWWE-3xFLAG	This study	RRID:Addgene_248319
pCAG-VPS13C-delVABdelATG2C-3xFLAG	This study	RRID:Addgene_248320
pCAG-VPS13C-VAB-3xFLAG	This study	RRID:Addgene_248321
pCMV10-VPS13C-WT-GFP-3xFLAG	This study	RRID:Addgene_248322
pCMV10-VPS13C-M392A W393D W395K I398D-GFP-3xFLAG	This study	RRID:Addgene_248323
pCMV10-VPS13C-W374K I378D L382K-GFP-3xFLAG	This study	RRID:Addgene_248324
pCMV10-VPS13C-W374K W393D W395K-GFP-3xFLAG	This study	RRID:Addgene_248325
pCMV10-VPS13C-W395C-GFP-3xFLAG	This study	RRID:Addgene_248326
pCMV10-VPS13C-delC-GFP-3xFLAG	This study	RRID:Addgene_248327
pCAG-Strep-Rab7-Q67L	This study	RRID:Addgene_248328
pCAG-MBP-TEV-Rab7-Q67L-6His	This study	RRID:Addgene_248329
pCAG-3xFLAG-CtVPS13	This study	RRID:Addgene_248330
pETDuet-6xHis-Strep-CtVPS13 (1-635)	This study	RRID:Addgene_248331
pETDuet-6xHis-Strep-CtVPS13 (1-635) + 6xHis-HsCaM	This study	RRID:Addgene_248332
pCMV10-mCherry-ATG2C (VPS13C)	Wang et al.^9^	RRID:Addgene_232868
pCMV10-VPS13C(1-1390)-mStayGold	This study	RRID:Addgene_255693
PPB-CAG-VPS13C^^^mStayGold	This study	RRID:Addgene_255695
CMV-R-GECO1.2	Wu et al.^36^	RRID:Addgene_45494
FLAG-VAPA	Huttlin et al.^37^	RRID:Addgene_255771
pETDuet-Rab7(Q67L)-6xHis	This study	RRID:Addgene_255527
pETDuet-Rab7(T22N)-6xHis	This study	RRID:Addgene_255528
Software and algorithms		
CryoSPARC Version 4.6.2	Punjani et al.^12^	https://cryosparc.com/;RRID:SCR_016501
ImageJ Version 1.52e	Schneider et al.^38^	https://imagej.net/ij/;RRID:SCR_003070
WinCoot 1.1.14	Casanal et al.^39^	https://www2.mrc-lmb.cam.ac.uk/personal/pemsley/coot/; RRID:SCR_014222
Phenix 1.21.2-5419	Afonine et al.^40^	https://phenix-online.org/; RRID:SCR_014224
ChimeraX 1.9	Goddard et al.^41^	https://www.rbvi.ucsf.edu/chimerax/; RRID:SCR_015872
SerialEM	Mastronarde^42^	https://bio3d.colorado.edu/SerialEM/; RRID:SCR_017293
AlphaFold 3	Abramson et al.^18^	https://alphafoldserver.com/about; RRID:SCR_025885
DeepMainMast	Terashi et al.^43^	https://em.kiharalab.org/algorithm/DeepMainMast
Namdinator	Kidmose et al.^44^	https://namdinator.au.dk/
spIsoNet	Liu et al.^45^	https://github.com/IsoNet-cryoET/spIsoNet
GraphPad Prism 6	GraphPad Prism	http://www.graphpad.com/; RRID:SCR_002798
PyMOL 2.6.0a0	Schrödinger, LLC	https://www.pymol.org/; RRID:SCR_000305
Custom Python script for Laurdan-GP analysis	This study; Zenodo	https://doi.org/10.5281/zenodo.19688024
Other		
Endogenous calmodulin co-purified with VPS13s detected by western blotting	This study; protocol.io	https://doi.org/10.17504/protocols.io.kqdg31w17l25/v1
Cell culture, transfection, immunocytochemistry, and imaging	Wang et al.^9^	https://doi.org/10.17504/protocols.io.eq2lyp55mlx9/v1
Expression, purification, and characterization of VPS13C	This study; protocol.io	https://doi.org/10.17504/protocols.io.rm7vz92d4gx1/v1
Cryo-EM structural determination of VPS13C	This study; protocol.io	https://doi.org/10.17504/protocols.io.36wgqpqpovk5/v1
Expression, purification, and characterization of CtVPS13(1-635) with calmodulin	This study; protocol.io	https://doi.org/10.17504/protocols.io.ewov11m17vr2/v1
Complex formation between VPS13C and Rab7	This study; protocol.io	https://doi.org/10.17504/protocols.io.eq2ly4q4qlx9/v1
Co-floatation assay of VPS13C with SUVs	This study; protocol.io	https://doi.org/10.17504/protocols.io.q26g7n9nqlwz/v1
Characterization of VPS13C’s binding to GUVs	This study; protocol.io	https://doi.org/10.17504/protocols.io.81wgbwko1gpk/v1
Stable Cell Line Generation	Bentley-DeSousa et al.^46^	https://doi.org/10.17504/protocols.io.yxmvm9nr5l3p/v1
Hydrogen-Deuterium Exchange Mass Spectrometry of the VPS13C-Calmodulin Complex	This study; protocol.io	https://doi.org/10.17504/protocols.io.q26g7o7q1vwz/v1

## Highlights:

A cryo-EM study reveals soluble VPS13C is in a lipid-transport inactive conformation.

In this conformation, its VAB domain blocks bridge domain membrane association.

Calmodulin bound at VPS13’s N-terminus is required for folding and localization.

Calmodulin complexed with VPS13C senses calcium levels.

## References

[1] Hanna M, Guillen-Samander A, and De Camilli P (2023). RBG Motif Bridge-Like Lipid Transport Proteins: Structure, Functions, and Open Questions. Annu Rev Cell Dev Biol 39, 409–434. 10.1146/annurev-cellbio-120420-014634. 37406299

[2] Reinisch KM, De Camilli P, and Melia TJ (2025). Lipid Dynamics at Membrane Contact Sites. Annu Rev Biochem 94, 479–502. 10.1146/annurev-biochem-083024-122821. 40067957

[3] Levine TP (2022). Sequence Analysis and Structural Predictions of Lipid Transfer Bridges in the Repeating Beta Groove (RBG) Superfamily Reveal Past and Present Domain Variations Affecting Form, Function and Interactions of VPS13, ATG2, SHIP164, Hobbit and Tweek. Contact (Thousand Oaks) 5, 251525642211343. 10.1177/25152564221134328. 36571082 PMC7613979

[4] Dziurdzik SK, and Conibear E (2021). The Vps13 Family of Lipid Transporters and Its Role at Membrane Contact Sites. Int J Mol Sci 22. 10.3390/ijms22062905. PMC7999203 33809364

[5] Kumar N, Leonzino M, Hancock-Cerutti W, Horenkamp FA, Li P, Lees JA, Wheeler H, Reinisch KM, and De Camilli P (2018). VPS13A and VPS13C are lipid transport proteins differentially localized at ER contact sites. J Cell Biol 217, 3625–3639. 10.1083/jcb.201807019. 30093493 PMC6168267

[6] Rampoldi L, Dobson-Stone C, Rubio JP, Danek A, Chalmers RM, Wood NW, Verellen C, Ferrer X, Malandrini A, Fabrizi GM, (2001). A conserved sorting-associated protein is mutant in chorea-acanthocytosis. Nat Genet 28, 119–120. 10.1038/88821. 11381253

[7] Ueno S, Maruki Y, Nakamura M, Tomemori Y, Kamae K, Tanabe H, Yamashita Y, Matsuda S, Kaneko S, and Sano A (2001). The gene encoding a newly discovered protein, chorein, is mutated in chorea-acanthocytosis. Nat Genet 28, 121–122. 10.1038/88825. 11381254

[8] Lesage S, Drouet V, Majounie E, Deramecourt V, Jacoupy M, Nicolas A, Cormier-Dequaire F, Hassoun SM, Pujol C, Ciura S, (2016). Loss of VPS13C Function in Autosomal-Recessive Parkinsonism Causes Mitochondrial Dysfunction and Increases PINK1/Parkin-Dependent Mitophagy. Am J Hum Genet 98, 500–513. 10.1016/j.ajhg.2016.01.014. 26942284 PMC4800038

[9] Wang X, Xu P, Bentley-DeSousa A, Hancock-Cerutti W, Cai S, Johnson BT, Tonelli F, Shao L, Talaia G, Alessi DR, (2025). The bridge-like lipid transport protein VPS13C/PARK23 mediates ER-lysosome contacts following lysosome damage. Nat Cell Biol 27, 776–789. 10.1038/s41556-025-01653-6. 40211074 PMC12081312

[10] Li P, Lees JA, Lusk CP, and Reinisch KM (2020). Cryo-EM reconstruction of a VPS13 fragment reveals a long groove to channel lipids between membranes. J Cell Biol 219. 10.1083/jcb.202001161. PMC7199853 32182622

[11] Hu B, Alvarez D, Rocha-Roa C, Guyard V, Li D, Wang X, De Camilli P, Vanni S, and Reinisch KM (2026). Molecular insights into bulk lipid transport from structural studies of the bridge-like protein VPS13A complexed with the scramblase XKR1 bioRxiv. 10.64898/2026.01.07.698282. PMC13267863 42285089

[12] Punjani A, Rubinstein JL, Fleet DJ, and Brubaker MA (2017). cryoSPARC: algorithms for rapid unsupervised cryo-EM structure determination. Nat Methods 14, 290–296. 10.1038/nmeth.4169. 28165473

[13] Jumper J, Evans R, Pritzel A, Green T, Figurnov M, Ronneberger O, Tunyasuvunakool K, Bates R, Zidek A, Potapenko A, (2021). Highly accurate protein structure prediction with AlphaFold. Nature 596, 583–589. 10.1038/s41586-021-03819-2. 34265844 PMC8371605

[14] Wang Y, Dahmane S, Ti R, Mai X, Zhu L, Carlson LA, and Stjepanovic G (2024). Structural basis for lipid transfer by the ATG2A-ATG9A complex. Nat Struct Mol Biol. 10.1038/s41594-024-01376-6. 39174844

[15] Kang Y, Lehmann KS, Long H, Jefferson A, Purice M, Freeman M, and Clark S (2025). Structural basis of lipid transfer by a bridge-like lipid-transfer protein. Nature 642, 242–249. 10.1038/s41586-025-08918-y. 40269155 PMC13533485

[16] Sakai Y, Matoba K, Kotani T, Hao L, Suzuki K, Kakuta C, Sugita Y, Osawa T, Nakatogawa H, N Noda N Mechanism of bridge-type phospholipid transfer by Atg2 for autophagosome biogenesis. (2025). bioRxiv. 10.1101/2025.05.24.655882

[17] Shen X, Valencia CA, Gao W, Cotten SW, Dong B, Huang BC, and Liu R (2008). Ca(2+)/Calmodulin-binding proteins from the C. elegans proteome. Cell Calcium 43, 444–456. 10.1016/j.ceca.2007.07.008. 17854888

[18] Abramson J, Adler J, Dunger J, Evans R, Green T, Pritzel A, Ronneberger O, Willmore L, Ballard AJ, Bambrick J, (2024). Accurate structure prediction of biomolecular interactions with AlphaFold 3. Nature 630, 493–500. 10.1038/s41586-024-07487-w. 38718835 PMC11168924

[19] Yan C, Wu F, Jernigan RL, Dobbs D, and Honavar V (2008). Characterization of protein-protein interfaces. Protein J 27, 59–70. 10.1007/s10930-007-9108-x. 17851740 PMC2566606

[20] Andrews C, Xu Y, Kirberger M, and Yang JJ (2020). Structural Aspects and Prediction of Calmodulin-Binding Proteins. Int J Mol Sci 22. 10.3390/ijms22010308. PMC7795363 33396740

[21] Kilmartin JV (2003). Sfi1p has conserved centrin-binding sites and an essential function in budding yeast spindle pole body duplication. J Cell Biol 162, 1211–1221. 10.1083/jcb.200307064. 14504268 PMC2173958

[22] De M, Oleskie AN, Ayyash M, Dutta S, Mancour L, Abazeed ME, Brace EJ, Skiniotis G, and Fuller RS (2017). The Vps13p-Cdc31p complex is directly required for TGN late endosome transport and TGN homotypic fusion. J Cell Biol 216, 425–439. 10.1083/jcb.201606078. 28122955 PMC5294781

[23] Smolders S, Philtjens S, Crosiers D, Sieben A, Hens E, Heeman B, Van Mossevelde S, Pals P, Asselbergh B, Dos Santos Dias R, (2021). Contribution of rare homozygous and compound heterozygous VPS13C missense mutations to dementia with Lewy bodies and Parkinson’s disease. Acta Neuropathol Commun 9, 25. 10.1186/s40478-021-01121-w. 33579389 PMC7881566

[24] Treiman M, Caspersen C, and Christensen SB (1998). A tool coming of age: thapsigargin as an inhibitor of sarco-endoplasmic reticulum Ca(2+)-ATPases. Trends Pharmacol Sci 19, 131–135. 10.1016/s0165-6147(98)01184-5. 9612087

[25] Gillingham AK, Bertram J, Begum F, and Munro S (2019). In vivo identification of GTPase interactors by mitochondrial relocalization and proximity biotinylation. Elife 8. 10.7554/eLife.45916. PMC6639074 31294692

[26] Gimenez-Andres M, Copic A, and Antonny B (2018). The Many Faces of Amphipathic Helices. Biomolecules 8. 10.3390/biom8030045. PMC6164224 29976879

[27] Gahlot P, Kravic B, Rota G, van den Boom J, Levantovsky S, Schulze N, Maspero E, Polo S, Behrends C, and Meyer H (2024). Lysosomal damage sensing and lysophagy initiation by SPG20-ITCH. Mol Cell 84, 1556–1569 e1510. 10.1016/j.molcel.2024.02.029. 38503285

[28] Vamparys L, Gautier R, Vanni S, Bennett WF, Tieleman DP, Antonny B, Etchebest C, and Fuchs PF (2013). Conical lipids in flat bilayers induce packing defects similar to that induced by positive curvature. Biophys J 104, 585–593. 10.1016/j.bpj.2012.11.3836. 23442909 PMC3566444

[29] Soczewka P, Kolakowski D, Smaczynska-de Rooij I, Rzepnikowska W, Ayscough KR, Kaminska J, and Zoladek T (2019). Yeast-model-based study identified myosin- and calcium-dependent calmodulin signalling as a potential target for drug intervention in chorea-acanthocytosis. Dis Model Mech 12. 10.1242/dmm.036830. PMC6361151 30635263

[30] Yeshaw WM, van der Zwaag M, Pinto F, Lahaye LL, Faber AI, Gomez-Sanchez R, Dolga AM, Poland C, Monaco AP, van ISC, (2019). Human VPS13A is associated with multiple organelles and influences mitochondrial morphology and lipid droplet motility. Elife 8. 10.7554/eLife.43561. PMC6389287 30741634

[31] Wardaszka P, Soczewka P, Sienko M, Zoladek T, and Kaminska J (2021). Partial Inhibition of Calcineurin Activity by Rcn2 as a Potential Remedy for Vps13 Deficiency. Int J Mol Sci 22. 10.3390/ijms22031193. PMC7865597 33530471

[32] Levine TP (2025). Update on VAP, a ubiquitous signpost for the ER. Biol Chem 406, 487–504. 10.1515/hsz-2025-0199. 41261728

[33] Guillen-Samander A, Leonzino M, Hanna MG, Tang N, Shen H, and De Camilli P (2021). VPS13D bridges the ER to mitochondria and peroxisomes via Miro. J Cell Biol 220. 10.1083/jcb.202010004. PMC8077184 33891013

[34] Valverde DP, Yu S, Boggavarapu V, Kumar N, Lees JA, Walz T, Reinisch KM, and Melia TJ (2019). ATG2 transports lipids to promote autophagosome biogenesis. J Cell Biol 218, 1787–1798. 10.1083/jcb.201811139. 30952800 PMC6548141

[35] Ashkenazy H, Abadi S, Martz E, Chay O, Mayrose I, Pupko T, and Ben-Tal N (2016). ConSurf 2016: an improved methodology to estimate and visualize evolutionary conservation in macromolecules. Nucleic Acids Res 44, W344–350. 10.1093/nar/gkw408. 27166375 PMC4987940

[36] Wu J, Liu L, Matsuda T, Zhao Y, Rebane A, Drobizhev M, Chang YF, Araki S, Arai Y, March K, (2013). Improved orange and red Ca(2)+/− indicators and photophysical considerations for optogenetic applications. ACS Chem Neurosci 4, 963–972. 10.1021/cn400012b. 23452507 PMC3689190

[37] Huttlin EL, Ting L, Bruckner RJ, Gebreab F, Gygi MP, Szpyt J, Tam S, Zarraga G, Colby G, Baltier K, (2015). The BioPlex Network: A Systematic Exploration of the Human Interactome. Cell 162, 425–440. 10.1016/j.cell.2015.06.043. 26186194 PMC4617211

[38] Schneider CA, Rasband WS, and Eliceiri KW (2012). NIH Image to ImageJ: 25 years of image analysis. Nat Methods 9, 671–675. 10.1038/nmeth.2089. 22930834 PMC5554542

[39] Casanal A, Lohkamp B, and Emsley P (2020). Current developments in Coot for macromolecular model building of Electron Cryo-microscopy and Crystallographic Data. Protein Sci 29, 1069–1078. 10.1002/pro.3791. 31730249 PMC7096722

[40] Afonine PV, Poon BK, Read RJ, Sobolev OV, Terwilliger TC, Urzhumtsev A, and Adams PD (2018). Real-space refinement in PHENIX for cryo-EM and crystallography. Acta Crystallogr D Struct Biol 74, 531–544. 10.1107/S2059798318006551. 29872004 PMC6096492

[41] Goddard TD, Huang CC, Meng EC, Pettersen EF, Couch GS, Morris JH, and Ferrin TE (2018). UCSF ChimeraX: Meeting modern challenges in visualization and analysis. Protein Sci 27, 14–25. 10.1002/pro.3235. 28710774 PMC5734306

[42] Mastronarde DN (2005). Automated electron microscope tomography using robust prediction of specimen movements. J Struct Biol 152, 36–51. 10.1016/j.jsb.2005.07.007. 16182563

[43] Terashi G, Wang X, Prasad D, Nakamura T, and Kihara D (2024). DeepMainmast: integrated protocol of protein structure modeling for cryo-EM with deep learning and structure prediction. Nat Methods 21, 122–131. 10.1038/s41592-023-02099-0. 38066344 PMC12815591

[44] Kidmose RT, Juhl J, Nissen P, Boesen T, Karlsen JL, and Pedersen BP (2019). Namdinator - automatic molecular dynamics flexible fitting of structural models into cryo-EM and crystallography experimental maps. IUCrJ 6, 526–531. 10.1107/S2052252519007619. PMC6608625 31316797

[45] Liu YT, Fan H, Hu JJ, and Zhou ZH (2025). Overcoming the preferred-orientation problem in cryo-EM with self-supervised deep learning. Nat Methods 22, 113–123. 10.1038/s41592-024-02505-1. 39558095 PMC12131231

[46] Bentley-DeSousa A, Ferguson S (2024). Stable Cell Line Generation – RAW 264.7. protocols.io. 10.17504/protocols.io.yxmvm9nr5l3p/v1.

[47] Morin A, Eisenbraun B, Key J, Sanschagrin PC, Timony MA, Ottaviano M, and Sliz P (2013). Collaboration gets the most out of software. Elife 2, e01456. 10.7554/eLife.01456. 24040512 PMC3771563

[48] Rosenthal PB, and Henderson R (2003). Optimal determination of particle orientation, absolute hand, and contrast loss in single-particle electron cryomicroscopy. J Mol Biol 333, 721–745. 10.1016/j.jmb.2003.07.013. 14568533

[49] Chen S, McMullan G, Faruqi AR, Murshudov GN, Short JM, Scheres SH, and Henderson R (2013). High-resolution noise substitution to measure overfitting and validate resolution in 3D structure determination by single particle electron cryomicroscopy. Ultramicroscopy 135, 24–35. 10.1016/j.ultramic.2013.06.004. 23872039 PMC3834153

[50] Song Y, DiMaio F, Wang RY, Kim D, Miles C, Brunette T, Thompson J, and Baker D (2013). High-resolution comparative modeling with RosettaCM. Structure 21, 1735–1742. 10.1016/j.str.2013.08.005. 24035711 PMC3811137

[51] Stariha JTB, Hoffmann RM, Hamelin DJ, and Burke JE (2021). Probing Protein-Membrane Interactions and Dynamics Using Hydrogen-Deuterium Exchange Mass Spectrometry (HDX-MS). Methods Mol Biol 2263, 465–485. 10.1007/978-1-0716-1197-5_22. 33877613

[52] Kong AT, Leprevost FV, Avtonomov DM, Mellacheruvu D, and Nesvizhskii AI (2017). MSFragger: ultrafast and comprehensive peptide identification in mass spectrometry-based proteomics. Nat Methods 14, 513–520. 10.1038/nmeth.4256. 28394336 PMC5409104

[53] da Veiga Leprevost F, Haynes SE, Avtonomov DM, Chang HY, Shanmugam AK, Mellacheruvu D, Kong AT, and Nesvizhskii AI (2020). Philosopher: a versatile toolkit for shotgun proteomics data analysis. Nat Methods 17, 869–870. 10.1038/s41592-020-0912-y. 32669682 PMC7509848

[54] Dobbs JM, Jenkins ML, and Burke JE (2020). Escherichia coli and Sf9 Contaminant Databases to Increase Efficiency of Tandem Mass Spectrometry Peptide Identification in Structural Mass Spectrometry Experiments. J Am Soc Mass Spectrom 31, 2202–2209. 10.1021/jasms.0c00283. 32869988

[55] Masson GR, Burke JE, Ahn NG, Anand GS, Borchers C, Brier S, Bou-Assaf GM, Engen JR, Englander SW, Faber J, (2019). Recommendations for performing, interpreting and reporting hydrogen deuterium exchange mass spectrometry (HDX-MS) experiments. Nat Methods 16, 595–602. 10.1038/s41592-019-0459-y. 31249422 PMC6614034

[56] Perez-Riverol Y, Bai J, Bandla C, Garcia-Seisdedos D, Hewapathirana S, Kamatchinathan S, Kundu DJ, Prakash A, Frericks-Zipper A, Eisenacher M, (2022). The PRIDE database resources in 2022: a hub for mass spectrometry-based proteomics evidences. Nucleic Acids Res 50, D543–D552. 10.1093/nar/gkab1038. 34723319 PMC8728295

[57] Parigoris E, Dunkelmann DL, and Silvan U (2020). Generation of Giant Unilamellar Vesicles (GUVs) Using Polyacrylamide Gels. Bio Protoc 10, e3807. 10.21769/BioProtoc.3807. PMC7842411 33659461

[58] Dodes Traian MM, Flecha FLG, and Levi V (2012). Imaging lipid lateral organization in membranes with C-laurdan in a confocal microscope. J Lipid Res 53, 609–616. 10.1194/jlr.D021311. 22184757 PMC3276485

[59] Kaiser HJ, Lingwood D, Levental I, Sampaio JL, Kalvodova L, Rajendran L, and Simons K (2009). Order of lipid phases in model and plasma membranes. Proc Natl Acad Sci U S A 106, 16645–16650. 10.1073/pnas.0908987106. 19805351 PMC2757813

[60] Gaus K, Zech T, and Harder T (2006). Visualizing membrane microdomains by Laurdan 2-photon microscopy. Mol Membr Biol 23, 41–48. 10.1080/09687860500466857. 16611579

[61] Owen DM, Rentero C, Magenau A, Abu-Siniyeh A, and Gaus K (2011). Quantitative imaging of membrane lipid order in cells and organisms. Nat Protoc 7, 24–35. 10.1038/nprot.2011.419. 22157973

