# Supplementary material for: Cryo-EM structure of soluble VPS13C suggests its regulation by a conformational switch and by calmodulin
Source: Mol Cell. Author manuscript; Available in PMC 2026 Aug 3. (PMC13430412; doi:10.1016/j.molcel.2026.06.028)
Supplement: 1 [file NIHMS2191195-supplement-1.pdf]

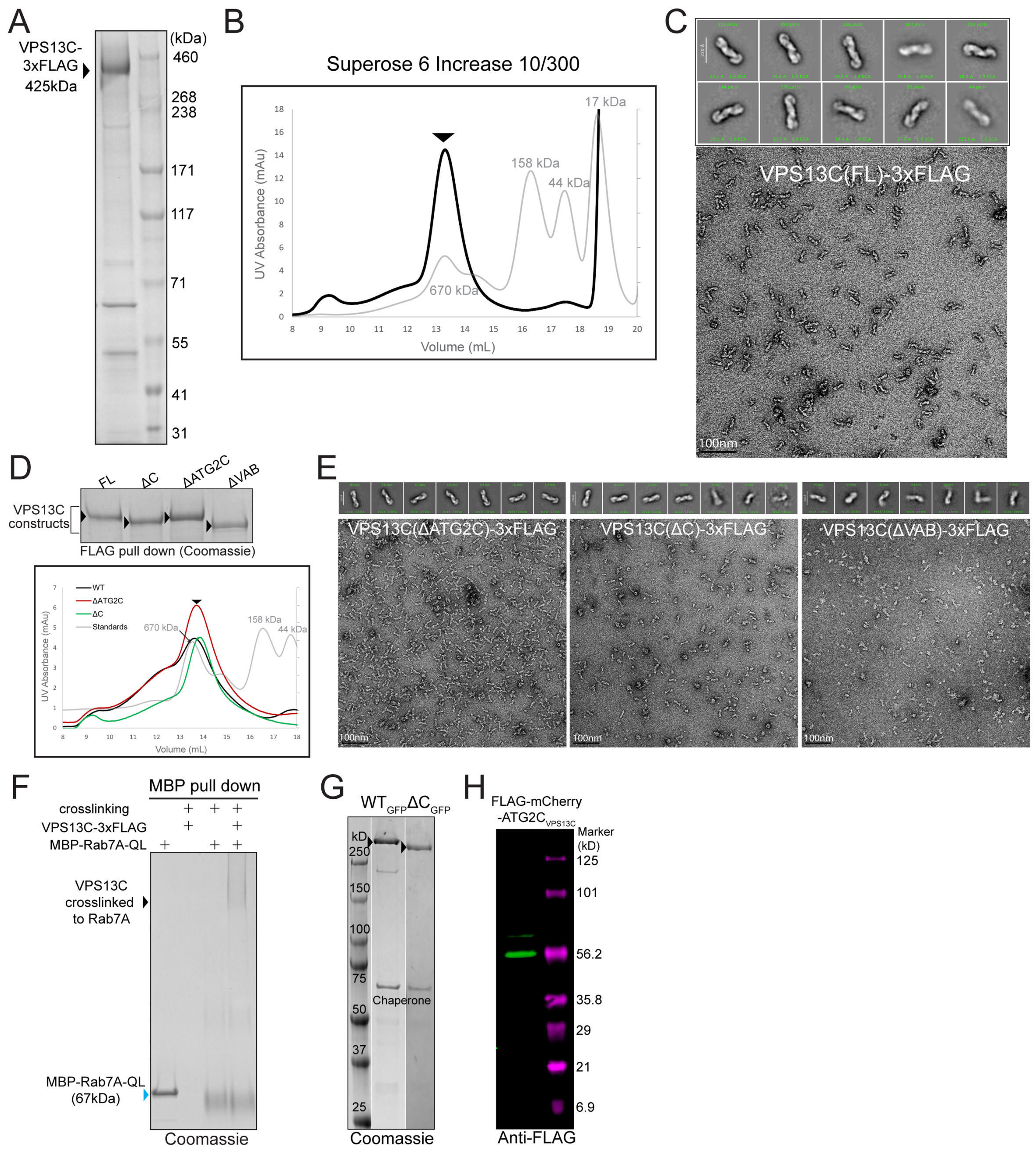

Figure S1. Preparation of VPS13C constructs for *in vitro* studies, related to Figure 1 and Figure 4. (**A**) Purified full-length VPS13C-3xFLAG expressed in Expi293F cells analyzed by SDS-PAGE. (**B**) Size-exclusion chromatography of VPS13C-3xFLAG (425 kDa) on a Superose 6 10/300 column shows a monodisperse peak eluting near the 670 kDa standard. (**C**) Representative negative-stain EM image and 2D averages reveal a homogeneous sample of full-length VPS13C with a rod-like shape. Scale bars, 100 nm. (**D**) VPS13C truncation mutants used for Rab7A pull down experiments and liposome flotation assays in Figure 4 are not degraded or aggregated, and migrate at their expected molecular weights on SDS-PAGE and on size-exclusion chromatography columns. (**E**) Representative negative-stain EM images and 2D averages show that VPS13C truncation mutants are well-folded and not aggregated. Scale bars, 100 nm. (**F**) Purified full-length FLAG-tagged VPS13C forms a complex with purified MBP-tagged Rab7A upon glutaldehyde crosslinking. The complex is shown as a high molecular-weight band on SDS-PAGE following MBP-IP. (**G**) GFP-tagged VPS13C WT and VPS13C-ΔC used for GUV experiments in Figure 4 are pure and intact, not degraded, as shown by SDS-PAGE. The white gap indicates where irrelevant lanes are digitally eliminated. (**H**) FLAG-mCherry-tagged VPS13C-ATG2C used in Figure 4 is intact in cell lysate, as shown by anti-FLAG western blot.

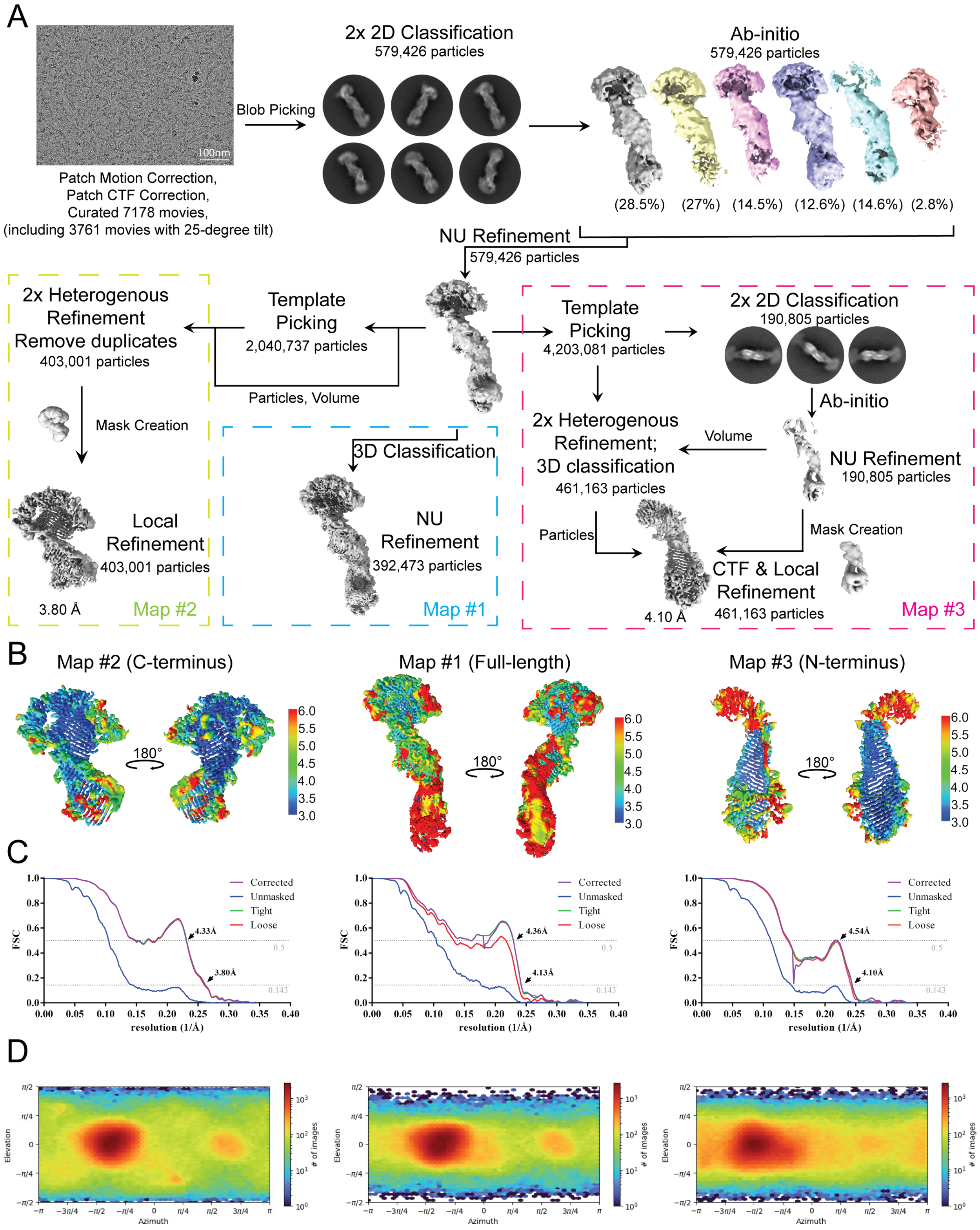

Figure S2. Data processing of full-length VPS13C-3xFLAG, related to Figure 1 and Table 1.
(**A**) Workflow of data processing. (**B**) Local resolution estimation of the three final maps, shown from front and back views. Map #1 is the lower-resolution full-length map; Map #2 and Map #3 are locally-refined maps of the C-terminus and the N-terminus, respectively. Maps are colored according to local resolutions (Å) (**C**) FSC curves from final refinements for all three maps. (**D**) Orientation distribution plots for particles contributing to the three maps, showing preferred orientation.

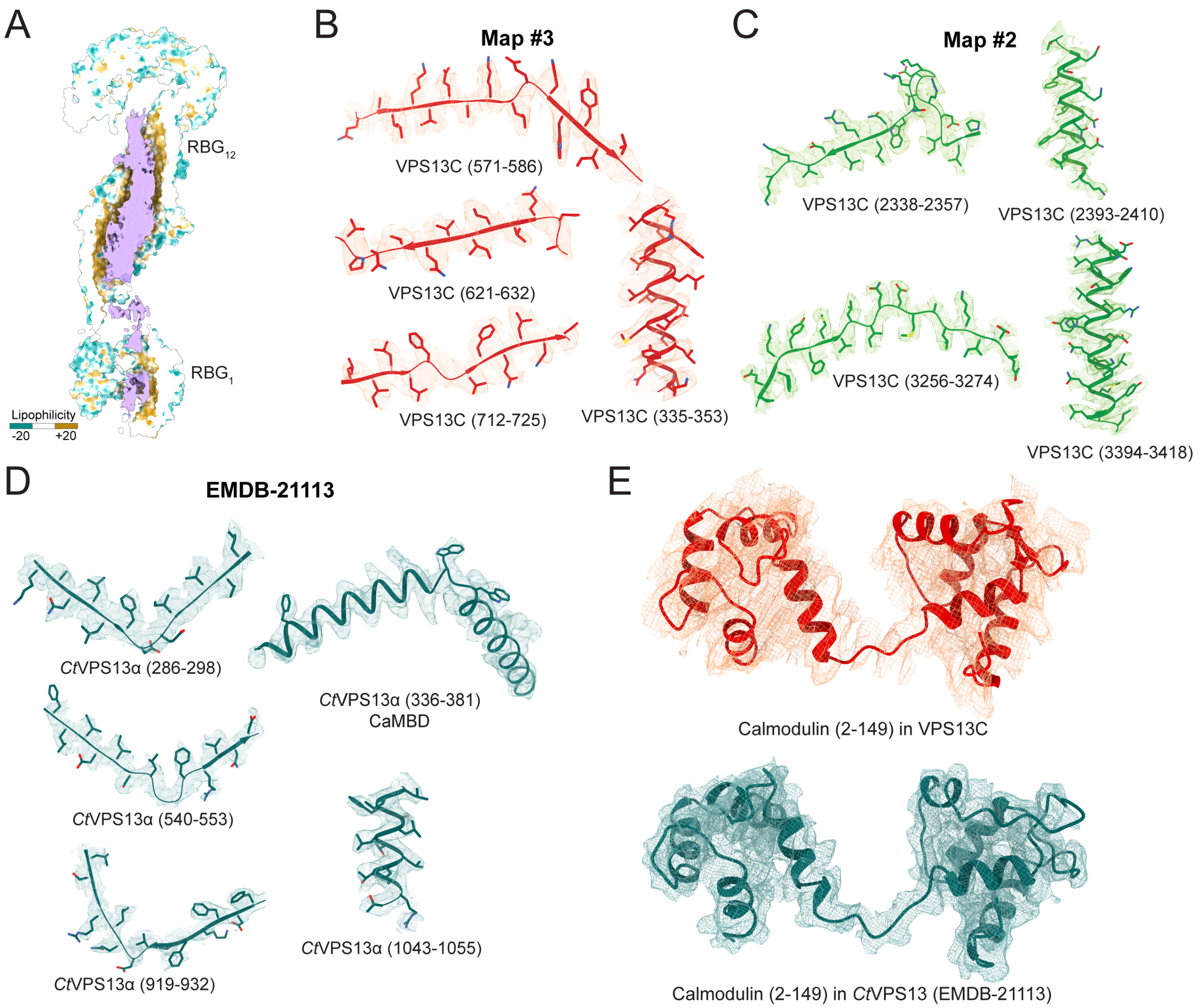

Figure S3. Map interpretation and model fitting for VPS13C and *Ct*VPS13α, related to Figure 1 and Table 1. (**A**) Cross-sectional view of the VPS13C model (colored by hydrophobicity) with residual EM density (purple) after subtraction of model-derived density. Density is shown at a low contour level. Continuous density is observed within the channel spanning RBG1 to RBG12 that can be accounted for by lipid occupancy. (**B–C**) Representative map–model fits for regions at the N-terminus (B) and C-terminus (C) of VPS13C. (**D**) Representative map–model fit for CtVPS13α (EMDB: 21113). (**E**) Calmodulin models fitted into human VPS13C (top) and CtVPS13α (bottom). Map-to-model correlation coefficients are 0.53 for VPS13C–CaM and 0.65 for CtVPS13α–CaM, consistent with the corresponding local resolutions (~5–6 Å for VPS13C and ~4–4.5 Å for CtVPS13α).

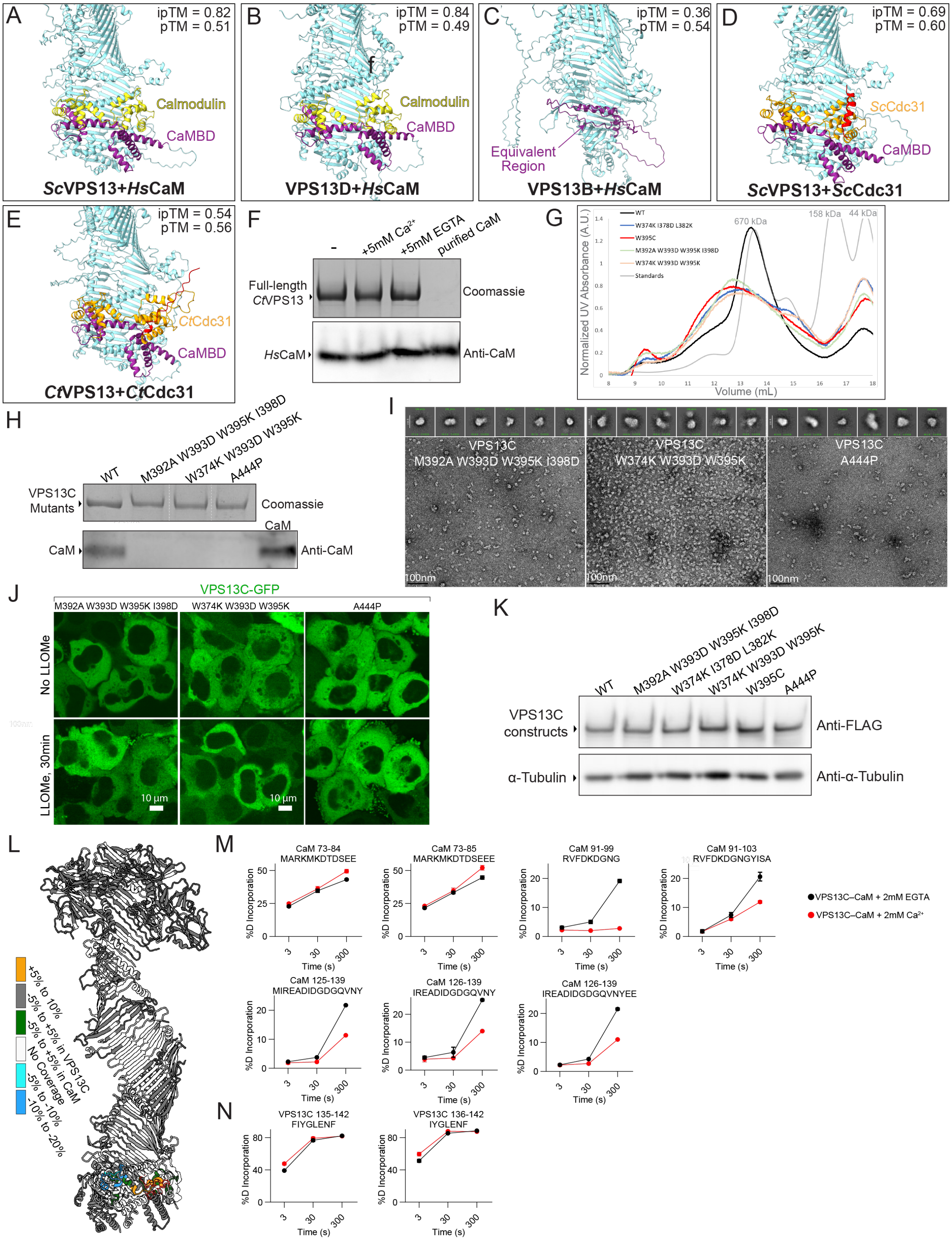
A
ipTM = 0.82
pTM = 0.51
Calmodulin
CaMBD
ScVPS13+HsCaM
B
ipTM = 0.84
pTM = 0.49
Calmodulin
CaMBD
VPS13D+HsCaM
C
ipTM = 0.36
pTM = 0.54
Equivalent Region
VPS13B+HsCaM
D
ipTM = 0.69
pTM = 0.60
ScCdc31
CaMBD
ScVPS13+ScCdc31
E
ipTM = 0.54
pTM = 0.56
CtCdc31
CaMBD
CtVPS13+CtCdc31
F
-
+5mM Ca2+
+5mM EGTA
purified CaM
Full-length CtVPS13
Coomassie
HsCaM
Anti-CaM
G
WT
W374K I378D L382K
W395C
M392A W393D W395K I398D
W374K W393D W395K
Standards
670 kDa
158 kDa
44 kDa
Normalized UV Absorbance (A.U.)
Volume (mL)
H
WT
M392A W393D W395K I398D
W374K W393D W395K
A444P
VPS13C Mutants
Coomassie
CaM
CaM
Anti-CaM
I
VPS13C M392A W393D W395K I398D
VPS13C W374K W393D W395K
VPS13C A444P
100nm
J
VPS13C-GFP
M392A W393D W395K I398D
W374K W393D W395K
A444P
No LLOMe
LLOMe, 30min
10 µm
K
WT
M392A W393D W395K I398D
W374K I378D L382K
W374K W393D W395K
W395C
A444P
VPS13C constructs
Anti-FLAG
α-Tubulin
Anti-α-Tubulin
L
+5% to 10%
-5% to +5% in VPS13C
-5% to +5% in CaM
No Coverage
-5% to -10%
-10% to -20%
M
CaM 73-84 MARKMKDTDSEE
CaM 73-85 MARKMKDTDSEEE
CaM 91-99 RVFDKDGNG
CaM 91-103 RVFDKDGNGYISA
CaM 125-139 MIREADIDGDGQVNY
CaM 126-139 IREADIDGDGQVNY
CaM 126-139 IREADIDGDGQVNYEE
%D Incorporation
Time (s)
VPS13C–CaM + 2mM EGTA
VPS13C–CaM + 2mM Ca2+
N
VPS13C 135-142 FIYGLENF
VPS13C 136-142 IYGLENF

Figure S4

Figure S4. Conservation, characterization, and calcium-dependent HDX-MS analysis of VPS13-CaM interactions, related to Figure 2. (**A**) Predicted interaction of Saccharomyces cerevisiae (*Sc*) VPS13 (light blue) with CaM (yellow) through a conserved CaM-binding domain (CaMBD, purple). (**B**) Predicted interaction of human VPS13D with CaM, colored as in (A). (**C**) Prediction of human VPS13B and CaM. The CaMBD-equivalent region between RBG_1 and RBG_2, is highlighted in purple. CaM is not predicted to interact with any part of VPS13B shown here. (**D**) Predicted interaction of *Sc*VPS13 with *Sc*Cdc31. *Sc*Cdc31 (orange) differs from human CaM by an additional N-terminal helix (red). (**E**) Predicted interaction of *Ct*VPS13 with *Ct*Cdc31. Similarly, *Ct*Cdc31 (orange) differs from human CaM by an additional N-terminal loop (red). (**F**) When overexpressed in Expi293F cells, 3xFLAG-*Ct*VPS13 co-purifies endogenous CaM in FLAG-IP. The binding of calmodulin to *Ct*VPS13 is unaffected by the presence of calcium or EGTA. (**G**) Size-exclusion chromatography of VPS13C WT and calmodulin-binding defective mutants. Mutants elute with broader and shifted peaks compared to WT, yet remain soluble and do not aggregate in the void volume. (**H**) Normalized FLAG-IP of 3xFLAG-tagged VPS13C mutants from Expi293F cells, shown by SDS-PAGE. The white dashed line indicates where irrelevant lanes are digitally eliminated. Co-purified endogenous CaM is detected by western blotting. Like the W395C VPS13C mutant (Fig. 2), VPS13C mutants A444P, M392A/W393D/W395K/I398D and W374K/W393D/W395K, which do not bind CaM, (**I**) no longer have the rod-like shape of the WT protein but remain non-aggregated, examined by negative-stain EM and 2D averaging (Scale bars, 100 nm), and (**J**) fail to localize to ER-lysosome contacts upon LLOME treatment in Hela cells. Scale bars, 10 µm. (**K**) Western blot of cell extracts showing that all CaM-binding defective mutants are expressed at levels comparable to VPS13C WT when overexpressed in Expi293F cells. (**L**) Peptides in the VPS13C–CaM complex that show significant HDX differences from EGTA to Ca^{2+} conditions (>0.4 Da and >5% change; two-tailed t-test, $p < 0.01$) are mapped onto the structure and colored as indicated. (**M-N**) %D graphs for all peptides in CaM (M) and VPS13C (N) that show significant changes between Ca^{2+} (red) and EGTA (black) conditions. Error bars represent standard deviations (n=3 for each time point).

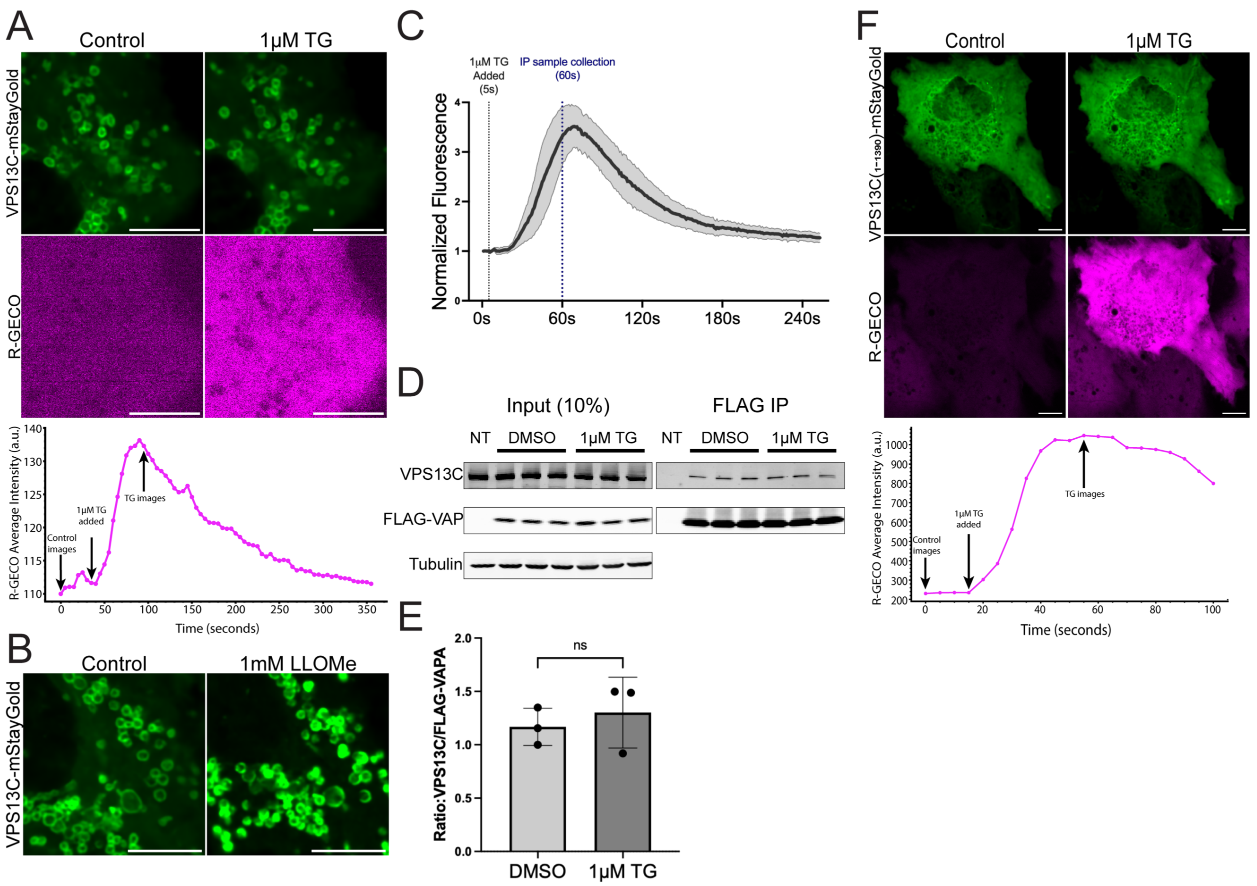

Figure S5. Effects of calcium elevation on VPS13C localization and interaction with VAP-A, related to Figure 2. (**A**) Thapsigargin-induced calcium elevation does not alter VPS13C localization. Representative fluorescence images of RPE-1 cells expressing VPS13C-mStayGold and calcium indicator R-GECO under basal conditions (control) or following treatment with 1 µM thapsigargin (TG). Images are stills taken from Video S1. Scale bars, 10 µm. The experiment was repeated three times with similar results. The graph below shows the quantification of mean R-GECO fluorescence intensity per frame from the field of view. Frames were acquired every 5 seconds. 1 µM thapsigargin was added at t = 35 seconds (frame 8, arrow). The timepoints corresponding to the captured images are indicated with arrows. a.u., arbitrary units. (**B**) LLOMe-induced lysosomal recruitment of VPS13C in RPE-1 cells. Representative fluorescence images of RPE-1 stably expressing VPS13C-mStayGold under basal conditions (control) or following treatment with 1mM LLOMe for 34 minutes. Images are taken from Video S2. Scale bars, 10 µm. The experiment was repeated three times with similar results. (**C**) Dynamics of cytosolic calcium measured by R-GECO fluorescence in HEK293T cells in response to 1 µM thapsigargin (TG) treatment. The average normalized fluorescence intensity was shown as a black line (n=17 cells), and the shaded region indicates ± standard deviation (s.d.). The blue dotted line indicates the time point at which samples were collected for experiments in (D) and (E). (**D**) FLAG-IP from HEK 293 cells expressing FLAG-VAPA. Treatment with 1 µM thapsigargin does not affect the amount of endogenous VPS13C co-immunoprecipitated with FLAG-VAPA. NT, non-transfected cells. Three biological replicates of each treatment are shown. The experiment was repeated twice with similar results. (**E**) Quantification of VPS13C co-immunoprecipitation in (D). Normalized ratios of co-immunoprecipitated VPS13C relative to FLAG-VAPA were shown as mean ± s.d. and compared using a two-sided Student's t-test. ns, not significant. (**F**) Thapsigargin-induced calcium elevation does not alter the localization of an N-terminal fragment of VPS13C. Representative fluorescence images of RPE-1 cells expressing N-terminal fragment $VPS13C_{(1-1390)}$-mStayGold and the calcium indicator R-GECO, under basal conditions (control) or following treatment with 1 µM thapsigargin (TG). Images are taken from Video S3. Scale bars, 10 µm. The experiment was repeated three times with similar results. The graph below shows the quantification of mean R-GECO fluorescence intensity per frame from the field of view. Frames were acquired every 5 seconds. 1 µM thapsigargin was added at t = 15 s (frame 4, arrow). The timepoints corresponding to the control and TG images are indicated with arrows. a.u., arbitrary units.

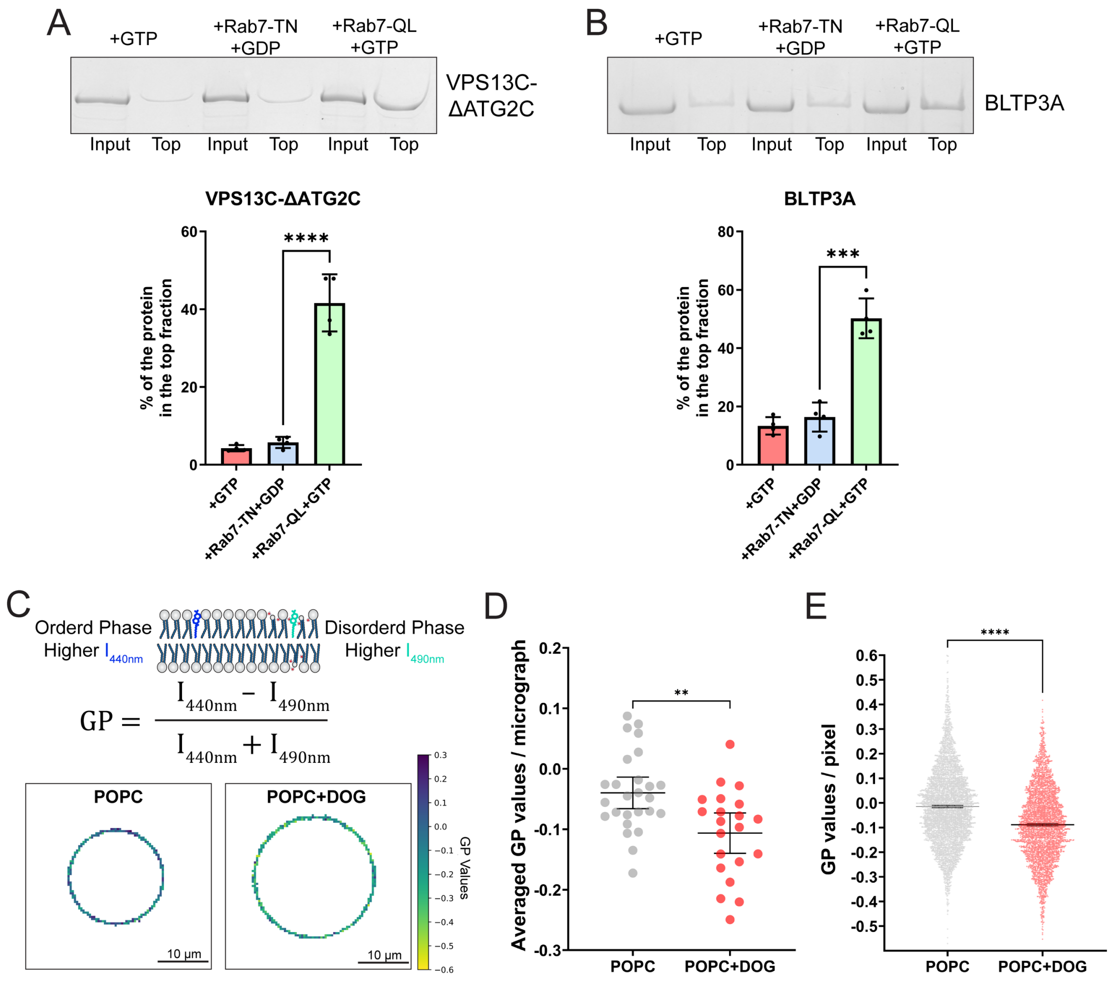

Figure S6. Rab7A nucleotide state and DOG-induced packing defects regulate VPS13C membrane recruitment, related to Figure 4. (**A**) VPS13C-ΔATG2C shows reduced membrane binding in liposome co-flotation assays. Recruitment of VPS13C-ΔATG2C is restored in the presence of the GTP-bound Rab7A mutant Q67L, but not by the GDP-bound/nucleotide-free mutant T22N. (**B**) Positive control experiment using purified BLTP3A, a known Rab7A effector.[1] BLTP3A associates with the membrane only in the presence of GTP-bound form of Rab7A (Q67L), but not with the GDP-bound/nucleotide-free form (T22N). The bar plots in (A) and (B) show the quantification of the fraction of VPS13C or BLTP3A that co-floated with liposomes, respectively; n=4. Data were shown as mean ± s.d. and compared using a two-sided Student's t-test; *****P*<0.0001; ****P*<0.001. (**C**) Schematic of the Laurdan generalized polarization (GP) experiment to confirm DOG-induced packing defects in GUVs. In tightly packed membranes (e.g., POPC), Laurdan exhibits an emission peak around 440 nm. In membranes with packing defects, increased water penetration shifts the emission peak toward 490 nm, resulting in lower GP values as calculated by the equation below. Representative images of POPC and POPC+DOG GUVs (bottom) are colored by GP values along the membrane surface. DOG-containing GUVs display overall lower GP values, consistent with increased membrane packing defects. Scale bars, 10 µm. (**D**) Quantification of mean GP values averaged across GUVs within each micrograph (n=26 for POPC; n=21 for POPC+DOG, where n represents each micrograph). (**E**) Pixel-wise distribution of GP values across all micrographs for both GUV conditions (n=4644 for POPC; n=3243 for POPC+DOG, where n represents each pixel). For both (D) and (E), data are presented as mean ± 95% confidence interval to indicate the precision of the estimated mean GP for each condition. Statistical comparisons were performed using Welch's t-test; $^{**}P < 0.01$; $^{****}P < 0.0001$.

Table S1. Plasmids used in this study, related to all figures.

No.	Name	Insert	Residue number and mutations	Addgene #
1	pCAG-VPS13C-WT-3xFLAG	VPS13C	1-3753	248315
2	pCAG-VPS13C-ΔATG2C-3xFLAG	VPS13C	1-3476-GS-3585-3753	248316
3	pCAG-VPS13C-ΔC-3xFLAG	VPS13C	1-3418	248317
4	pCAG-VPS13C-ΔVAB-3xFLAG	VPS13C	1-2411-3x(GGGGS)-3068-3753	248318
5	pCAG-VPS13C-ΔCΔWWE-3xFLAG	VPS13C	1-3116; 3185-3418	248319
6	pCAG-VPS13C-ΔATG2CΔVAB-3xFLAG	VPS13C	1-2411-3x(GGGGS)-3068-3476-GS-3585-3753	248320
7	pCAG-VPS13C-VAB-3xFLAG	VPS13C	2422-3083	248321
8	pCMV10-VPS13C-WT-GFP-3xFLAG	VPS13C	1-3753	248322
9	pCMV10-VPS13C-M392A W393D W395K I398D-GFP-3xFLAG	VPS13C	1-3753(M392A W393D W395K I398D)	248323
10	pCMV10-VPS13C-W374K I378D L382K-GFP-3xFLAG	VPS13C	1-3753(W374K I378D L382K)	248324
11	pCMV10-VPS13C-W374K W393D W395K-GFP-3xFLAG	VPS13C	1-3753(W374K W393D W395K)	248325
12	pCMV10-VPS13C-W395C-GFP-3xFLAG	VPS13C	1-3753(W395C)	248326
13	pCMV10-VPS13C-ΔC-GFP-3xFLAG	VPS13C	1-3418	248327
14	pCMV10-mCherry-ATG2C	VPS13C	3419-3609	232868
15	pCAG-Strep-Rab7-Q67L	Rab7A	1-207 (Q67L)	248328
16	pCAG-MBP-TEV-Rab7-Q67L-6His	Rab7A	1-207 (Q67L)	248329
17	pCAG- 3xFLAG-*Ct*VPS13	*Ct*VPS13	1-3225	248330
18	pETDuet-6xHis-Strep-*Ct*VPS13 (1-635)	*Ct*VPS13	1-635	248331
19	pETDuet-6xHis-Strep-*Ct*VPS13 (1-635) + 6xHis-*Hs*CaM	*Ct*VPS13, *Hs*CaM	1-635; 2-149	248332
20	pCMV10-VPS13C(1-1390)-mStayGold	VPS13C	1-1390	255693
21	CMV-R-GECO1.2	R-GECO1.2	Synthetic	45494
22	PPB-CAG-VPS13C^mStayGold	VPS13C	1-3753	255695
23	FLAG-VAPA	VAP-A	1-249	255771
24	pET29-Rab7(Q67L)-6xHis	Rab7A	1-207 (Q67L)	255527
25	pET29-Rab7(T22N)-6xHis	Rab7A	1-207 (T22N)	255528

1. Hanna, M.G., Rodriguez Cruz, H.O., Fujise, K., Wu, Y., Xu, C.S., Pang, S., Li, Z., Monetti, M., and De Camilli, P. (2025). BLTP3A is associated with membranes of the late endocytic pathway and is an effector of CASM. EMBO J *44*, 6168-6195. 10.1038/s44318-025-00543-9.
